# Genotype-phenotype correlations of fasting C-peptide and lipids in HNF1A-MODY: a single-center series and multi-center cross-sectional analysis in Chinese population

**DOI:** 10.3389/fendo.2026.1735596

**Published:** 2026-02-12

**Authors:** Mengyu Wang, Hulian Huang, Hualin Liu, Huihui Tian, Xinguo Hou, Li Chen, Meng Tian, Lingshu Wang

**Affiliations:** 1 Department of Endocrinology and Metabolism, Qilu Hospital of Shandong University, Jinan, Shandong, China; 2 The First Clinical Medical College, Cheeloo College of Medicine, Shandong University, Jinan, Shandong, China; 3 Jinan Aixin Zhuoer Medical Testing Co., Ltd, Jinan, Shandong, China; 4 Shandong Provincial Key Laboratory of Spatiotemporal Regulation and Precision Intervention in Endocrine and Metabolic Diseases, Shandong Provincial Engineering Research Center for Advanced Technologies in Prevention and Treatment of Chronic Metabolic Diseases, Institute of Endocrine and Metabolic Diseases of Shandong University, Jinan, Shandong, China; 5 Department of Endocrinology, Weihai Municipal Hospital, Weihai, Shandong, China

**Keywords:** clinical characteristics, genotype-phenotype correlation, HNF1A mutations, maturity-onset diabetes of the young, type 3

## Abstract

**Background:**

HNF1A-MODY is one of the most prevalent subtypes of maturity-onset diabetes of the young (MODY). Individuals with HNF1A-MODY display considerable clinical heterogeneity, potentially attributable to specific mutation sites. However, in the Chinese population, the relationship between distinct mutation sites and clinical manifestations remains to be investigated.

**Methods:**

In the initial analysis, 23 HNF1A-MODY patients diagnosed at the Department of Endocrinology, Qilu Hospital were included. These patients were followed up regularly to monitor glycemic control status and the progression of complications. In the subsequent analysis, baseline information of 113 Chinese HNF1A-MODY retrieved from public databases were further enrolled. Analysis of covariance was conducted to investigate the genotype-phenotype associations.

**Results:**

This study included a total of 136 patients. Among the 23 from Qilu Hospital, 22 distinct *HNF1A* gene variants were identified, including 8 novel ones. After excluding cases classified as “variant of uncertain significance”, the analysis showed that the median age of onset was earliest in patients with DNA-binding domain mutations (15.70 years), compared to the dimerization or transactivation domain mutations (*p* = 0.044). Fasting C-peptide levels were markedly lower in the dimerization domain and DNA-binding domain group (*p* = 0.005). Patients with DNA-binding domain mutations demonstrated lower low-density lipoprotein cholesterol (*p* = 0.049) and total cholesterol (*p* = 0.016) levels, but higher high-density lipoprotein cholesterol (*p* = 0.036) levels. Analysis of covariance indicated that mutations in the dimerization domain (mean difference = -0.757, *p* = 0.001) and DNA-binding domain (mean difference = -0.331, *p* = 0.041) were independently associated with lower fasting C-peptide, and DNA-binding domain mutations were also associated with low-density lipoprotein cholesterol (mean difference = -0.554, *p* = 0.015) and higher high-density lipoprotein cholesterol (mean difference = 0.224, *p* = 0.015) levels, whereas the other domain mutations showed no statistically significant associations.

**Conclusion:**

This study revealed the correlation between *HNF1A* mutation regions and pancreatic islet function as well as blood lipids in Chinese HNF1A-MODY patients, thereby underscoring the importance of early genetic identification in formulating individualized therapeutic strategies to improve prognosis.

## 1 Introduction

Maturity-onset diabetes of the young (MODY) is a monogenic form of diabetes characterized by autosomal dominant inheritance. To date, 14 distinct MODY subtypes have been identified, among which HNF1A-MODY, caused by *HNF1A* gene mutations, represents one of the most prevalent subtypes, accounting for 30-70% of all MODY cases (1). However, recent study indicates that within the Chinese MODY population, the proportion of HNF1A-MODY cases is 16.03% (2). The *HNF1A* gene encodes hepatocyte nuclear factor 1α (HNF1α), a transcriptional regulator that activates insulin gene expression in pancreatic β-cells. Structurally, HNF1α consists of three functional domains (3): an N-terminal dimerization domain (residues 1-32), a DNA-binding domain (residues 91-281), and a C-terminal transactivation domain (residues 282-631). HNF1A-MODY is characterized by defective insulin secretion and progressive hyperglycemia, with a high prevalence of microvascular complications (4). The clinical phenotypes of HNF1A-MODY patients exhibit considerable heterogeneity and overlap with both type 1 diabetes mellitus (T1DM) and type 2 diabetes mellitus (T2DM) (5), often leading to misdiagnosis. Therefore, early and accurate genetic diagnosis followed by precision therapy is essential for improving clinical management and long-term outcomes.

The marked heterogeneity in the clinical manifestations of HNF1A-MODY may be attributed to the variability in mutation loci (6). Previous studies have indicated that both the type and location of *HNF1A* mutations influence the age at diagnosis (7, 8), response to sulfonylurea therapy (9), and risk of diabetic complications (10). Additionally, genetic modifiers and exposure to intrauterine hyperglycemia may contribute to the variability in HNF1A-MODY diagnosis age (11, 12). However, the current researches were mainly based on data from Caucasian and other non-Chinese Asian populations, while systematic analyses of Chinese HNF1A-MODY patients remain scarce. Additionally, the impact of domain-specific *HNF1A* mutations on metabolic indicators such as pancreatic function and lipids has not been thoroughly investigated.

This study analyzed patients with HNF1A-MODY admitted to Qilu Hospital of Shandong University, along with reported Chinese cases from published literature, to investigate the genotype-phenotype correlations associated with domain-specific *HNF1A* mutations. This study aims to provide a basis for developing personalized management strategies by investigating the association between specific mutation sites and patients’ clinical manifestations, thereby improving the prognosis of *HNF1A* mutation carriers.

## 2 Materials and methods

### 2.1 Subjects

This study included patients from two sources. First, we enrolled 23 HNF1A-MODY patients with *HNF1A* gene variants identified through gene sequencing, who were treated at the Department of Endocrinology, Qilu Hospital of Shandong University from January 2021 to April 2025. These patients were followed up regularly to monitor their glycemic control status and the progression of complications. Fasting plasma glucose < 7 mmol/L and glycated hemoglobin < 6.5% are defined as good glycemic control (4), whereas failure to meet either criterion indicates poor glycemic control.

Additionally, 113 patients were identified through a review of the literature. Using the keywords and their Chinese equivalents, including “Maturity-Onset Diabetes of the Young, Type 3”, “MODY, Type 3”, “MODY3”, “HNF1A-MODY”, “HNF1-alpha MODY”, “hepatic nuclear factor 1 alpha”, “HNF1A”, and “HNF1-alpha”, a comprehensive literature search was conducted in databases including China National Knowledge Infrastructure (CNKI), Wanfang Data Knowledge Service Platform, China Science and Technology Journal Database (CQVIP), Chinese Medical Journal Network (CMJN), PubMed and Web of Science to identify relevant literature on Chinese patients with HNF1A-MODY. The search period was set from inception of the database to April 30, 2025. The detailed search strategy is provided in **Supplementary Appendix S1**.

**Figure 1:**
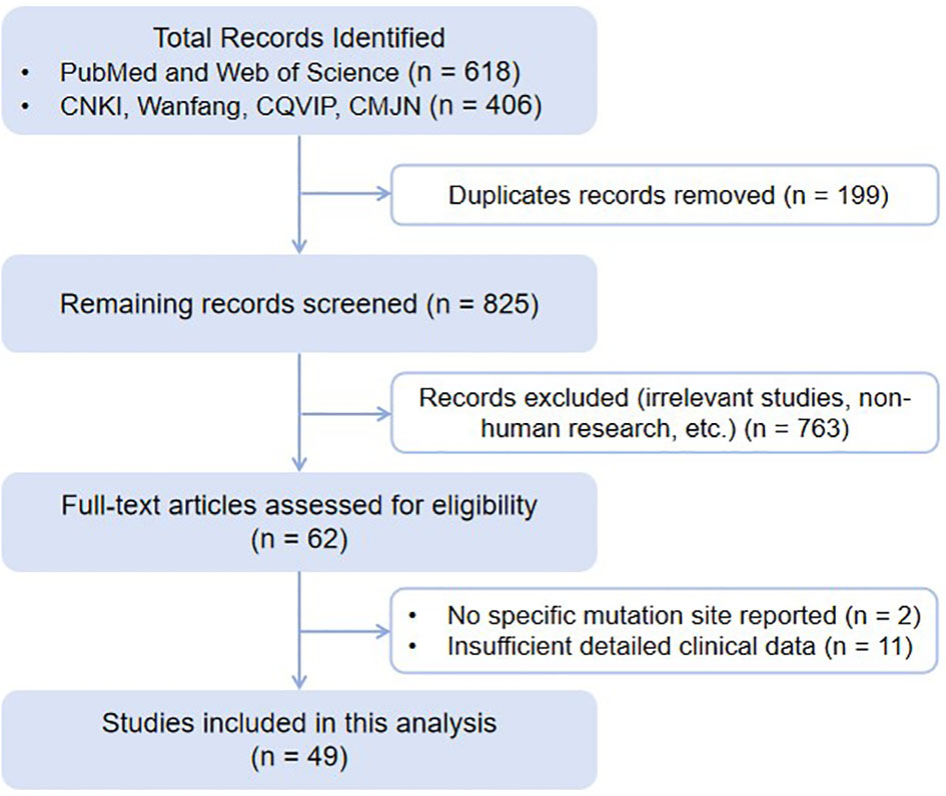
Literature flow diagram.

Literature screening followed the PRISMA guidelines. Initially, duplicates were removed using EndNote software, followed by a manual review of authors and affiliations to exclude duplicate reports. A two-stage screening was then performed: firstly, titles and abstracts were reviewed to exclude obviously irrelevant studies, non-human research, and non-original case reports (e.g., reviews, commentaries). Secondly, full texts of the initially screened articles were reviewed, and studies were selected for inclusion according to predetermined inclusion and exclusion criteria. Finally, we examined the participants’ dates of birth reported by the included studies. Cases were screened based on date of birth and sex to identify and exclude duplicate subjects. Inclusion Criteria: (1) Chinese HNF1A-MODY patient was confirmed by gene sequencing; (2) explicitly reported the location and type of *HNF1A* mutation; (3) reported at least three of the following parameters: age of onset, fasting plasma glucose (FPG), 2-hour postprandial glucose (2hPG), fasting C-peptide (FCP), 2-hour postprandial C-peptide (2hPCP), glycated hemoglobin (HbA_1c_), lipid profile, antidiabetic therapies, or diabetic complications. Exclusion Criteria: (1) unavailable or overlapping data; (2) lack of mutation information or key clinical characteristics. The screening process is presented in a PRISMA flowchart (**Figure 1**).

### 2.2 Clinical data collection

Clinical data, including the gender, age of onset, age of diagnosis, duration of diabetes, presence of classic diabetes symptoms (including polydipsia, polyuria, polyphagia, and unexplained weight loss — the presence of any one of these symptoms is considered indicative of classic hyperglycemic symptoms (13).), family history, history of smoking and drinking, body mass index (BMI), FPG, 2hPG, fasting insulin (FINS), 2-hour postprandial insulin (2hPINS), FCP (measured by chemiluminescence immunoassay after an 8-hour fasting period), 2hPCP, HbA_1c_, islet autoantibody (including IAA, ICA, IA-2A, and GAD), triglyceride (TG), total cholesterol (TC), high-density lipoprotein cholesterol (HDL-C), low-density lipoprotein cholesterol (LDL-C), uric acid (UA), hypersensitive C-reactive protein (hs-CRP), glucosuria, diabetic complications — diabetic retinopathy is screened through fundus examination, diabetic peripheral neuropathy is screened via nerve conduction velocity and vibration perception threshold testing and macrovascular complications are assessed using carotid ultrasound, history of underlying diseases (hypertension, coronary heart disease, etc.), antidiabetic therapies, history of other medications (including lipid-lowering therapy, etc.), and the location and type of *HNF1A* mutation were systematically collected. To ensure comparability across data sources, variables with inconsistent measurement units were standardized prior to pooled analysis.

### 2.3 Statistical analysis

Statistical analysis was performed using SPSS 27.0 software. Normally distributed numerical data were expressed as mean ± standard deviation and were compared using independent sample t-test or one-way ANOVA, followed by Bonferroni-adjusted *post-hoc* test. Non-normally distributed data were presented as M (Q1, Q3), and compared using the Mann-Whitney U test or Kruskal-Wallis H test, with *post-hoc* pairwise comparisons conducted using Dunn’s test. Categorical data were expressed as n (%), and group comparisons were performed using the chi-square test or Fisher’s exact test. To investigate whether different mutation domains are independently associated with specific clinical manifestations after adjusting for potential confounding factors, analysis of covariance (ANCOVA) was employed. To control for variation across different data sources, we incorporated data source as a random effect and performed analyses using linear mixed model. A *p*-value < 0.05 was considered statistically significant.

## 3 Results

### 3.1 Baseline characteristics of HNF1A-MODY patients in Qilu cohort

This study enrolled 23 patients diagnosed with HNF1A-MODY at Qilu Hospital (**Tables 1**, **2**). Whole-exome sequencing identified 22 distinct *HNF1A* variants, including 8 novel ones, which were classified as pathogenic, likely pathogenic, or variant of uncertain significance according to the standards and guidelines recommended by the American College of Medical Genetics (ACMG) (**Table 1**). In terms of domain distribution, 2 variants were located in the dimerization domain, 9 in the DNA-binding domain, and 12 in the transactivation domain. Mutations were most frequently located in exons 2 and 4, accounting for 43.48% (10/23). The main variant types were missense (52.17%, 12/23) and frameshift mutations (26.09%, 6/23). Clinically (**Table 2**), all patients presented with early-onset diabetes characteristic of HNF1A-MODY, with an age of onset ranging from 9 to 29 years, all < 35 years. Most patients (81.82%, 18/22) had a BMI < 24 kg/m^2^. A positive family history of diabetes was reported in 82.61% (19/23) of cases. At disease onset, 43.48% (10/23) of the patients manifested classic symptoms of polydipsia, polyuria, polyphagia, weight loss. 2 patients tested positive for islet autoantibodies. Regarding complications, 40.91% (9/22) had developed at least one diabetic complication, with diabetic peripheral neuropathy (36.36%, 8/22) and diabetic retinopathy (22.72%, 5/22) being the most common. In terms of glucose-lowering regimens, 8.70% (2/23) of patients were managed with lifestyle intervention alone; 56.52% (13/23) received oral antidiabetic drug monotherapy; and the remaining 34.78% (8/23) required insulin, with 8.70% (2/23) on insulin monotherapy and 26.08% (6/23) on a combination of oral agents and insulin. Given that the pathogenicity of novel variants had not yet been functionally validated, we excluded cases classified as “variant of uncertain significance” from subsequent analyses. A comparison of the clinical characteristics among different mutation domains was provided in **Supplementary Table 2**.

**Table 1:** The genotypes of patients with HNF1A-MODY in Qilu Hospital.

Patient	Mutation site	Domain	REVEL	PolyPhen	MutationTaster	SIFT	GERP	Source of variation	Reference^b^	ACMG classification
1	c.130dup, p. Leu44ProfsTer16	Dimerization domain	/	/	/	/	/	Maternal	(36)	Pathogenic
2	c.139G>A, p. Gly47Arg	Dimerization domain	0.572	0.011	0.663	0.062	3.31	Maternal	(37)	Uncertain
3	c.335del, p. Pro112ArgfsTer43^a^	DNA-binding domain	/	/	/	/	/	Paternal		Likely pathogenic
4	c.335del, p. Pro112ArgfsTer43^a^	DNA-binding domain	/	/	/	/	/	Paternal		Likely pathogenic
5	c.401_413delTCGATACCACTGG, p. Val134AlafsTer17^a^	DNA-binding domain	/	/	/	/	/	Paternal		Likely pathogenic
6	c.607C>G, p. Arg203Gly	DNA-binding domain	0.888	0.842	1	0	2.67	Maternal	(38)	Pathogenic
7	c.685C>T, p. Arg229Ter	DNA-binding domain	/	/	1	/	2.27	Maternal	(39)	Pathogenic
8	c.713 + 10C>T, -	DNA-binding domain	/	/	/	/	/	NA		Uncertain
9	c.811C>T, p. Arg271Trp	DNA-binding domain	0.93	0.993	1	0	3.94	*De novo*	(40)	Pathogenic
10	c.814C>T, p. Arg272Cys	DNA-binding domain	0.941	0.979	1	0.001	3.88	Maternal	(41)	Pathogenic
11	c.815G>A, p. Arg272His	DNA-binding domain	0.961	0.954	1	0	4.84	Paternal	(42, 43)	Pathogenic
12	c.872dup, p. Gly292ArgfsTer25	Transactivation domain	/	/	/	/	/	Paternal	(43)	Pathogenic
13	c.901G>A, p. Ala301Thr	Transactivation domain	0.645	0.056	0.999	0.496	3.77	NA		Uncertain
14	c.932C>A, p. Ala311Asp	Transactivation domain	0.593	0.138	1	0.014	4.67	NA	(44)	Uncertain
15	c.955G>C, p. Gly319Arg^a^	Transactivation domain	0.821	0.748	0.999	0.002	4.67	Maternal		Uncertain
16	c.994dupG, p. Glu322GlyfsTer87^a^	Transactivation domain	/	/	/	/	/	Maternal		Likely pathogenic
17	c.1135C>A, p. Pro379Thr	Transactivation domain	0.967	1	1	0	4.63	Maternal	(7)	Likely pathogenic
18	c.1349_1384del, p. Asn450Val462delinsIle^a^	Transactivation domain	/	/	/	/	/	*De novo*		Likely pathogenic
19	c.1502-6G>A, p.?	Transactivation domain	/	/	/	/	/	Maternal	(45)	Pathogenic
20	c.1604G>A, p. Ser535Asn^a^	Transactivation domain	0.361	0.036	0.573	0.582	5.52	NA		Uncertain
21	c.1673C>T, p. Pro558Leu	Transactivation domain	0.484	0.155	1	0.002	5.8	Maternal	(46)	Uncertain
22	c.1757C>A, p. Ala586Asp^a^	Transactivation domain	0.265	0.034	0.992	0.018	4.8	Paternal		Uncertain
23	c.1769-9C>A, splicing^a^	Transactivation domain	/	/	/	/	/	Paternal		Uncertain

a: novel variants, b: previously reported as HNF1A-MODY.

**Table 2:** Clinical and laboratory data of patients with HNF1A-MODY in Qilu hospital.

Patient	Sex	Age of onset (years)	BMI (kg/m^2^)	Family history	FPG (mmol/L)	2hPG (mmol/L)	FCP (ng/ml)	2hPCP(ng/ml)	Islet autoantibody	HbA_1c_ (%)	TG (mmol/L)	TC (mmol/L)	HDL-C (mmol/L)	LDL-C (mmol/L)	Complications	Treatment
1	F	17	21.36	yes	5.43	12.73	0.85	3.57	–	8.50	1.75	4.25	1.26	2.51	none	DPP-4i+AGI
2	F	16	17.31	no	14.94	19.50	0.41	1.08	–	17.80	0.39	6.08	2.04	3.39	none	Metformin+INS
3	F	25	23.62	yes	8.70	13.54	1.49	5.96	–	7.40	0.79	3.66	1.20	2.34	DPN	DPP-4i+AGI
4	F	29	18.83	yes	5.72		1.31		–	6.20	0.75	4.12	1.84	2.06	none	DPP-4i
5	M	16	22.68	yes	4.57	23.74	1.34	2.54	–	11.60	0.90	2.33	1.03	0.95	DPN,NPDR	Glimepiride+Metformin+DPP-4i
6	F	9	20.41	yes	12.90		1.02		–	8.90	0.80	4.20	1.34	1.80	none	INS
7	F	11	18.88	yes	11.40	16.90	1.30	2.65	–	8.80	0.88	4.12	1.32	2.54	none	Glimepiride
8	M	27	20.42	yes	4.97	15.44	0.27	2.07	–	10.70	0.74	3.10	1.17	1.64	Macrovascular disease,DPN	Gliquidone+Metformin
9	F	21	18.51	yes	8.15	23.43	0.62	1.47	IA-2Ab (+)	12.28	0.46	4.11	1.52	2.09	NPDR	Glimepiride
10	F	11	20.68	yes	4.74	12.66	0.82	1.55	–	9.00	0.83	2.31	1.16	0.90	DKA,DPN,NPDR	Gliquidone+Metformin+INS
11	F	10	18.07	yes	7.03	9.88	0.65	2.94	–	9.30	0.74	4.09	1.57	2.03	none	Glimepiride
12	M	11	20.00	yes	21.00	18.00	2.17	3.50	–	9.80	0.65	3.46	1.55	1.59	none	Gliquidone+DPP-4i
13	M	15	27.76	yes	7.00		2.70		–	7.00	1.89	5.08	1.02	3.71	none	Gliquidone+Metformin
14	M	26	27.04	yes	15.00		1.03		–	9.00	2.50	5.80	1.01	3.68		Gliquidone+Metformin+DPP-4i +INS
15	M	13	20.52	yes	6.27	12.50	1.45	4.00	–	6.40	0.92	4.01	1.14	2.67	none	INS
16	F	12	19.13	yes	4.20	10.52	0.51	1.85	–	8.00	0.65	3.22	1.12	1.76	DPN	Gliquidone+Metformin
17	F	25	27.55	yes	6.45	10.10	1.63	4.24	–	5.90	0.75	4.67	1.25	2.90	none	Lifestyles
18	M	15	18.28	yes	7.72	11.3	2.07	4.27	–	6.80	0.78	3.31	1.12	1.97	none	DPP-4i
19	M	11		yes	5.12	11.19	1.71	5.57	–	6.70	0.63	3.89	1.38	2.22	none	Lifestyles
20	M	24	28.08	no	7.65	13.63	1.61	1.58	–	12.30	1.93	4.49	0.90	3.10	DPN,NPDR	Glimepiride+Metformin+SGLT-2i+INS
21	M	11	22.89	no	9.22	24.09	0.02		–	12.30	1.02	4.80	1.41	3.12	DPN,DR	Metformin+INS
22	M	17	22.22	no	6.77	21.00	1.77	2.01	GAD (+)	11.30	0.74	3.73	0.76	2.61	none	Metformin+AGI+INS
23	M	25	23.54	yes	7.72	7.61	1.65	5.41	–	7.50	0.73	3.34	0.83	2.14	DPN	Metformin

BMI, body mass index; FPG, fasting plasma glucose; 2hPG, 2-hour postprandial blood glucose; FCP, fasting C-peptide; 2hPCP, 2-hour postprandial C-peptide; HbA1c, glycated hemoglobin; TG, triglyceride; TC, total cholesterol; HDL-C, high-density lipoprotein cholesterol; LDL-C, low-density lipoprotein cholesterol; NPDR, non-proliferative diabetic retinopathy; DPN, diabetic peripheral neuropathy; DPP-4i, dipeptidyl peptidase 4 inhibitor; AGI, alpha glucosidase inhibitor; INS, insulin; SGLT-2i, sodium-glucose transporter 2 inhibitor.

### 3.2 Follow-up data of HNF1A-MODY patients at Qilu hospital

**Figure 2:**
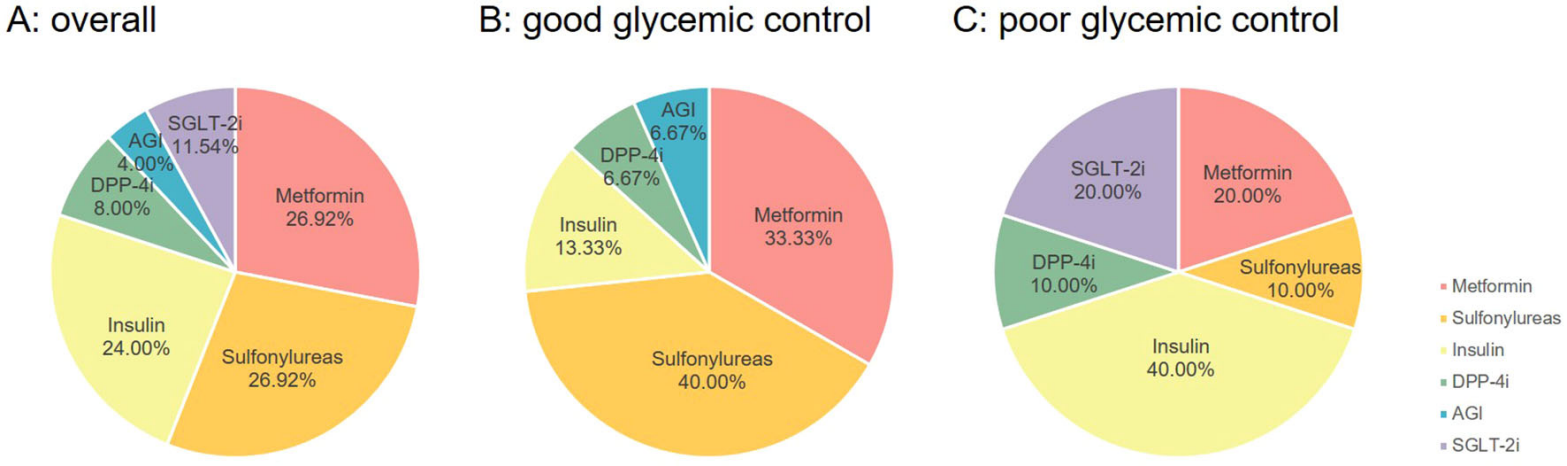
Analysis of hypoglycemic drugs for HNF1A-MODY patients. **(A)** all types of hypoglycemic drugs for patients with HNF1A-MODY, **(B)** the proportion of hypoglycemic drugs in HNF1A-MODY patients with good glycemic control, **(C)** the proportion of hypoglycemic drugs in HNF1A-MODY patients with poor glycemic control. Abbreviations: DPP-4i, dipeptidyl peptidase 4 inhibitor; AGI, alpha glucosidase inhibitor; SGLT-2i, sodium-glucose transporter 2 inhibitor.

During a median follow-up of 2 years, 2 patients were lost to follow-up. Among the remaining 21 patients, 71.43% (15/21) achieved good glycemic control, defined as FPG < 7 mmol/L and HbA_1c_ < 6.5% (4). Within this limited observation window, two new cases of diabetic peripheral neuropathy and one case of diabetic retinopathy were documented. After excluding cases classified as “variant of uncertain significance”, the analysis of hypoglycemic regimens (**Figure 2**) showed that sulfonylureas were the most common medication among patients with good glycemic control (40.00%), whereas insulin usage was highest in the poor control group. Comparative analysis based on glycemic control status (**Table 3**) demonstrated that the poor glycemic control group had higher baseline BMI (*p* = 0.010) and LDL-C (*p* = 0.042) than the good control group. Regarding hypoglycemic therapy, the good glycemic control group was mainly managed with oral hypoglycemic monotherapy (*p* = 0.025), while the poor control group more frequently required insulin-based combination therapy (*p* = 0.039). No significant intergroup differences were observed in age of onset, mutation domain distribution, blood glucose, HbA_1c_, or C-peptide levels at baseline.

**Table 3:** Baseline clinical characteristics of patients with HNF1A-MODY by glycemic control status.

Subject	Good glycemic control (n=10)	Poor glycemic control (n=5)	*P*-value
Age of onset (years)	14.60 ± 6.26	18.80 ± 6.46	0.247
Dimerization domain (%)	1/10 (10.0)	1/5 (20.0)	0.591
DNA-binding domain (%)	6/10 (60.0)	1/5 (20.0)	0.143
Transactivation domain (%)	3/10 (30.0)	3/5 (60.0)	0.264
Classic diabetes symptoms (%)	7/10 (70.0)	2/5 (40.0)	0.264
BMI (kg/m^2^)	19.42 ± 1.12	23.49 ± 4.13	0.010*
FPG (mmol/L)	7.38 (5.26, 11.78)	9.22 (5.51, 14.97)	0.594
2hPG (mmol/L)	14.43 ± 4.65	19.36 ± 6.51	0.158
FCP (ng/ml)	1.13 ± 0.59	0.89 ± 0.66	0.475
2hPCP (ng/ml)	2.73 ± 1.03	2.62 ± 1.58	0.898
HbA_1c_ (%)	8.76 ± 1.66	11.32 ± 4.41	0.120
TG (mmol/L)	0.77 (0.65, 0.84)	0.90 (0.57, 1.76)	0.371
TC (mmol/L)	3.72 ± 0.63	4.74 ± 1.48	0.050
HDL-C (mmol/L)	1.38 ± 0.24	1.35 ± 0.42	0.851
LDL-C (mmol/L)	1.93 ± 0.47	2.81 ± 1.08	0.042*
UA (umol/L)	304.00 (298.00, 309.00)	365.00 (280.00, 450.00)	1.000
Diabetic kidney disease (%)	0	0	
Diabetic peripheral neuropathy (%)	2/10 (20.0)	2/4 (50.0)	0.262
Diabetic retinopathy (%)	2/10 (20.0)	2/4 (50.0)	0.262
Microvascular complications (%)	0	0	
Lifestyles (%)	0	1/5 (20.0)	0.143
OHA (%)	8/10 (80.0)	1/5 (20.0)	0.025*
INS (%)	1/10 (10.0)	0	0.464
OHA+INS (%)	1/10 (10.0)	3/5 (60.0)	0.039*

Classic diabetes symptoms, polydipsia, polyuria, polyphagia, weight loss; BMI, body mass index; FPG, fasting plasma glucose; 2hPG, 2-hour postprandial blood glucose; FCP, fasting C-peptide; 2hPCP, 2-hour postprandial C-peptide; HbA1c, glycated hemoglobin; TG, triglyceride; TC, total cholesterol; HDL-C, high-density lipoprotein cholesterol; LDL-C, low-density lipoprotein cholesterol; UA, uric acid; OHA, oral hypoglycemic drugs; INS, insulin; *: *p* < 0.05.

### 3.3 Clinical manifestations of Chinese HNF1A-MODY patients

Following the description of clinical manifestations from the Qilu Hospital cohort, we performed a detailed genotype-phenotype analysis in Chinese patients with HNF1A-MODY. Given that the pathogenicity of novel variants had not yet been functionally validated, we excluded cases classified as “variant of uncertain significance” from subsequent analyses. After a comprehensive literature review and exclusion of cases with incomplete clinical data or duplicate reports, baseline information of 113 HNF1A-MODY patients from published literature were included. Combined with the baseline data of the Qilu Hospital cohort (**Table 4**), the median age of onset was 19.00 years in the dimerization domain group, 15.70 years in the DNA-binding domain group, and 22.00 years in the transactivation domain group, indicating an earlier disease onset in patients with DNA-binding domain mutations (*p* = 0.044). Pancreatic islet function varied by domain. FCP levels were markedly lower in in the dimerization domain (1.00 ± 0.41 ng/ml) and DNA-binding domain group (1.08 ± 0.57 ng/ml) than the transactivation domain group (1.51 ± 0.85 ng/ml, *p* = 0.005). 2hPCP levels in the DNA-binding domain group [2.54 (1.56, 3.13) ng/ml] were also significantly lower than in the transactivation domain group [3.61 (1.83, 5.72) ng/ml, *p* = 0.041]. Lipid profiles also showed significant differences. HDL-C levels were higher in the DNA-binding domain group (1.45 ± 0.46 mmol/L, *p* = 0.036) than in the dimerization domain (1.27 ± 0.47 mmol/L) and transactivation domain group (1.20 ± 0.30 mmol/L). Conversely, the levels of LDL-C (DNA-binding domain group 2.47 ± 0.79 mmol/L vs. dimerization domain group 2.96 ± 0.46 mmol/L vs. transactivation domain group 2.98 ± 0.96 mmol/L, *p* = 0.049) and TC (DNA-binding domain group 4.15 ± 0.98 mmol/L vs. dimerization domain group 4.99 ± 0.75 mmol/L vs. transactivation domain group 4.93 ± 1.37 mmol/L, *p* = 0.016) showed different patterns. Regarding hypoglycemic therapy, patients with dimerization and transactivation domain variants were more frequently managed with diet and exercise alone (*p* = 0.008), while oral hypoglycemic agents were most common in the DNA-binding domain group (61.3%, *p* = 0.019). No significant differences were observed among groups in classic diabetes symptoms, BMI, FPG, 2hPG, HbA_1c_, TG, UA, insulin resistance indices, islet autoantibody, family history, or diabetic chronic complications.

**Table 4:** Clinical characteristics of Chinese HNF1A-MODY patients by different domains.

Subject	Total (n=130)	Dimerization domain (n=15)	DNA-binding domain (n=62)	Transactivation domain (n=53)	*p*-value
Age of onset (years)	17.92 (12.86, 25.00)	19.00 (16.00, 23.00)	15.70 (12.00, 24.00)	22.00 (13.00, 30.50)	0.044*
Classic diabetes symptoms (%)	45/93 (48.4)	8/15 (53.3)	23/40 (57.5)	14/38 (36.8)	0.173
BMI (kg/m^2^)	21.46 ± 3.26	21.74 ± 4.46	20.77 ± 2.50	22.21 ± 3.53	0.060
Family history (%)	117/130 (90.0)	12/15 (80.0)	57/62 (91.9)	48/53 (90.6)	0.378
FPG (mmol/L)	7.48 (6.00, 9.00)	7.40 (5.43, 10.60)	7.50 (6.09, 8.60)	7.48 (5.91, 9.11)	0.942
2hPG (mmol/L)	15.73 ± 5.37	16.42 ± 5.18	16.07 ± 5.56	15.23 ± 5.35	0.731
FCP (ng/ml)	1.25 ± 0.71	1.00 ± 0.41	1.08 ± 0.57	1.51 ± 0.85^ab^	0.005*
2hPCP (ng/ml)	2.77 (1.57, 4.25)	3.03 (1.49, 4.10)	2.54 (1.56, 3.13)	3.61 (1.83, 5.72)^b^	0.123
Islet autoantibody	3/124 (2.4)	0	1/59 (1.7)	2/51 (3.9)	0.617
HbA_1c_ (%)	8.00 (6.80, 9.75)	8.25 (6.73, 13.05)	8.10 (6.90, 9.55)	7.55 (6.65, 9.65)	0.244
HOMA-IR	2.71 (1.44, 3.96)	3.44 (1.53, 3.93)	3.38 (2.25, 4.38)	2.84 (1.86, 5.58)	0.648
TyG	8.99 ± 0.77	9.20 ± 0.72	8.86 ± 0.77	9.12 ± 0.77	0.290
TyG-BMI	185.25 (157.13, 210.44)	167.36 (146.16, 236.95)	183.60 (156.87, 205.38)	188.29 (157.77, 236.47)	0.295
HOMA-β (ins)	37.05 (23.22, 92.14)	34.03 (23.72, 147.49)	33.19 (23.60, 89.12)	41.23 (17.67, 99.47)	0.974
HOMA-β (CP)	1.75 (0.83, 3.63)	2.64 (1.03, 3.77)	2.60 (1.91, 3.75)	2.99 (1.16, 4.44)	0.074
TG (mmol/L)	1.15 (0.79, 1.79)	1.59 (0.74, 3.23)	1.02 (0.80, 1.54)	1.18 (0.78, 2.22)	0.584
TC (mmol/L)	4.56 ± 1.20	4.99 ± 0.75	4.15 ± 0.98	4.93 ± 1.37^b^	0.016*
HDL-C (mmol/L)	1.33 ± 0.41	1.27 ± 0.47	1.45 ± 0.46	1.20 ± 0.30^b^	0.036*
LDL-C (mmol/L)	2.75 ± 0.88	2.96 ± 0.46	2.47 ± 0.79	2.98 ± 0.96	0.049*
UA (umol/L)	299.77 ± 78.37	272.00 ± 50.91	313.63 ± 60.53	289.76 ± 94.96	0.597
Diabetic kidney disease (%)	22/129 (17.1)	0	11/62 (17.7)	11/52 (21.2)	0.155
Diabetic peripheral neuropathy (%)	23/129 (17.8)	1/15 (6.7)	10/62 (16.1)	12/52 (23.1)	0.305
Diabetic retinopathy (%)	26/129 (20.2)	0	16/62 (25.8)	10/52 (19.2)	0.080
Microvascular complications (%)	5/129 (3.9)	0	1/62 (1.6)	4/52 (7.7)	0.175
Lifestyles (%)	16/130 (12.3)	4/15 (26.7)	2/62 (3.2)	10/53 (18.9)	0.008*
OHA (%)	63/130 (48.5)	6/15 (40.0)	38/62 (61.3)	19/53 (35.8)	0.019*
INS (%)	25/130 (19.2)	2/15 (13.3)	14/62 (22.6)	9/53 (17.0)	0.620
OHA+INS (%)	26/130 (20.0)	3/15 (20.0)	8/62 (12.9)	15/53 (28.3)	0.120
lipid-lowering therapy	10/108 (9.3)	0	7/53 (13.2)	3/42 (7.1)	0.282

Classic diabetes symptoms, polydipsia, polyuria, polyphagia, weight loss; BMI, body mass index; FPG, fasting plasma glucose; 2hPG, 2-hour postprandial blood glucose; FCP, fasting C-peptide; 2hPCP, 2-hour postprandial C-peptide; HbA1c, glycated hemoglobin; HOMA-IR, homeostasis model assessment of insulin resistance; TyG, triglyceride-glucose; TyG-BMI, triglyceride-glucose-body mass index; HOMA-β (ins), homeostasis model assessment β cell function (based on Insulin); HOMA-β (CP), homeostasis model assessment β cell function (based on C-Peptide); TG, triglyceride; TC, total cholesterol; HDL-C, high-density lipoprotein cholesterol; LDL-C, low-density lipoprotein cholesterol; UA, uric acid; OHA, oral hypoglycemic drugs; INS, insulin; a: Compared with the dimerization domain, *p* < 0.05; b: Compared with the DNA-binding domain, *p* < 0.05; *: *p* < 0.05.

### 3.4 DNA-binding domain mutations independently associated with lower FCP, LDL-C and higher HDL-C levels

Our previous findings demonstrated that FCP levels were significantly lower in the dimerization domain and DNA-binding domain groups compared to the transactivation domain group. LDL-C and TC levels in the DNA-binding domain group were also significantly lower than those in the other two groups, while HDL-C was the opposite. To investigate whether the effects of mutation domains were independent of potential confounding factors, we enrolled an additional control group of 50 adolescents with type 2 diabetes who were randomly selected and conducted ANCOVA. The results showed that after adjusting for age of onset, gender, family history, duration of diabetes, BMI, HbA_1c_, TyG index, and prior antidiabetic therapies, the mutation domain remained an independent influencing factor for FCP levels. Mutations in the dimerization domain (mean difference = -0.757, *p* = 0.001) and DNA-binding domain (mean difference = -0.331, *p* = 0.041) were independently associated with lower FCP levels, while the effect of the transactivation domain did not reach statistical significance (**Table 5A**). In addition, after adjusting for age of onset, gender, history of smoking and drinking, BMI, HbA_1c_, TG, and lipid-lowering therapy, DNA-binding domain mutations were associated with lower LDL-C levels (mean difference = -0.554, *p* = 0.015) (**Supplementary Table 3**). DNA-binding domain mutations were also associated with higher HDL-C levels (mean difference = 0.224, *p* = 0.015) after adjusting for age of onset, gender, history of smoking and drinking, HbA_1c_, TG, TC, and lipid-lowering therapy. (**Table 5B**). In contrast, DNA-binding domain mutations were not independent influencing factors for lower TC levels (**Supplementary Table 3**).

**Table 5A:** Analysis of covariance of FCP levels in HNF1A-MODY patients.

Dependent variable	Domain	Mean difference	*P*-value	95% Confidence Interval	
				Lower	Upper
FCP (ng/ml)	dimerization domain	-0.757	0.001*	-1.205	-0.309
	DNA-binding domain	-0.331	0.041*	-0.647	-0.014
	transactivation domain	-0.063	0.698	-0.387	0.260

*: *p* < 0.05, after adjusting for age of onset, gender, family history, duration of diabetes, body mass index, glycated hemoglobin, TyG index, and prior antidiabetic therapies.

**Table 5B:** Analysis of covariance of HDL-C levels in HNF1A-MODY patients.

Dependent variable	Domain	Mean difference	*P*-value	95% Confidence Interval	
				Lower	Upper
HDL-C (mmol/L)	dimerization domain	0.111	0.468	-0.193	0.416
	DNA-binding domain	0.224	0.015*	0.045	0.403
	transactivation domain	0.075	0.366	-0.089	0.238

*: *p* < 0.05, after adjusting for age of onset, gender, history of smoking and drinking, glycated hemoglobin, triglyceride, total cholesterol, and lipid-lowering therapy.

Given the high proportion of insulin-treated patients across the included studies, to verify model stability, we further excluded clinically atypical cases with “early or sustained insulin dependence” (insulin initiation within 10 years of disease duration) and repeated the ANCOVA. The results showed that dimerization domain and DNA-binding domain mutations remained associated with lower FCP, and DNA-binding domain mutations continued to be linked to higher HDL-C levels (**Supplementary Table 4**).

Furthermore, we constructed linear mixed models with FCP, LDL-C, and HDL-C as outcome variables. The mutation domains were included as fixed effects, while data source was incorporated as a random intercept to account for variability across sources. After controlling for variation among different data sources, the mutation domain retained a statistically significant association with FCP, LDL-C, and HDL-C (**Supplementary Table 5**).

## 4 Discussion

This study analyzed the clinical characteristics of Chinese patients with HNF1A-MODY and revealed associations between mutations in different functional domains and clinical phenotypes. This multicenter cross-sectional analysis of Chinese population revealed that patients with DNA-binding domain variants exhibited a significantly younger age of onset, as well as lower FCP, LDL-C, TC levels, and higher HDL-C levels compared to patients with dimerization or transactivation domain mutations. Short-term follow-up revealed that HNF1A-MODY patients with poor glycemic control might had higher baseline BMI and LDL-C levels. ANCOVA indicated that DNA-binding domain mutations were independently associated with lower FCP, LDL-C and higher HDL-C levels.

HNF1A-MODY is a monogenic form of diabetes caused by pathogenic variants in the *HNF1A* gene. The *HNF1A* gene, located on chromosome 12q24 (14), encodes the hepatocyte nuclear factor 1 alpha (HNF1α) protein, which is predominantly expressed in the liver, pancreas, kidney, and gastrointestinal tract (15). HNF1α plays a critical role in regulating genes involved in insulin secretion, glucose metabolism, and lipid homeostasis. To date, approximately 500 *HNF1A* variants have been reported in association with MODY phenotypes (16). The *HNF1A* gene exhibits high polymorphism, with the highest mutation rate in exons 2 and 4 (17, 18). A retrospective study of patients with HNF1A-MODY reported 492 different mutations (10), and missense mutations (227, 46.1%) and frameshift mutations (198, 40.2%) were the most common mutations. Whereas in the Qilu Hospital cohort, 22 distinct *HNF1A* gene variants were identified, eight of which were novel, mainly located in exons 2 and 4, with missense and frameshift mutations being the main types, consistent with the previous findings.

The HNF1α protein comprises three functional domains: the dimerization domain mediates homodimerization or heterodimerization with homologous proteins such as HNF1β (19), which is fundamental for DNA-binding and transcriptional regulation, and aberrant assembly of the dimeric complex leads to impaired target gene binding and reduced transactivation activity. The DNA-binding domain consists of a POU-specific domain (POUS) and a POU homeodomain (POUH). POUS maintains protein stability, and POUH is critical for initiating protein-DNA interactions at promoter regions of target genes (20). The nuclear localization of HNF1α depends on specific nuclear localization signals, with residues 158-171, 197-205, and 271–282 are considered to be nuclear localization regions (21), mutations affecting these regions may lead to loss of transcriptional regulation. The transactivation domain recruits coactivators such as p300/CBP to activate the transcription of glucose metabolism-related genes (22). In our study, the number of cases in the dimerization domain group was relatively small, which may be attributed to the overall low frequency of mutations in this domain across populations. A retrospective study on HNF1A-MODY patients reported that (10) in non-Asian populations, mutation rates were approximately 7.8% in the dimerization domain, 29.2% in the DNA-binding domain, and 47.5% in the transactivation domain. Among Asian populations, the corresponding proportions were 3.8%, 48.8%, and 47.5%, with no significant difference in domain-specific mutation distribution between the two groups. Of the 432 patients with coding−region mutations for whom mutation sites were reported, only 15 occurred in the dimerization domain. In our Qilu Hospital cohort, mutations were observed in 8.7% of cases within the dimerization domain, 39.1% within the DNA-binding domain, and 52.2% within the transactivation domain, consistent with previous reports.

Previous researches demonstrated that the DNA-binding domain of HNF1α plays a more critical role in regulating blood glucose, and the dimerization domain plays the second most important role (17), indicating a possible genotype-phenotype correlation in HNF1A-MODY. To explore the association between genotype and clinical phenotypes in Chinese HNF1A-MODY patients, our study enrolled a total of 136 patients with from our cohort, together with previously reported cases from China identified through a review of published literature. Combined analysis with literature-sourced HNF1A-MODY cases revealed that patients with the dimerization and DNA-binding domain mutations presented a younger age of onset and lower FCP levels. Besides, patients with DNA-binding domain mutation exhibited significantly lower 2hPCP levels than those with transactivation domain mutation, suggesting that DNA-binding domain mutation cause more severe insulin secretion defects. This clinical difference indicates that mutations in the transactivation domain may lead to a milder diabetic phenotype. The impact of mutations varies by domain: mutations in the dimerization or DNA-binding domain impair DNA-binding capacity (23) and may cause more severe insulin secretion defects, resulting in a younger age of onset and lower C-peptide levels. The transactivation domain demonstrates greater tolerance to mutations that result in small changes in HNF1α protein structure than the dimerization or DNA-binding domain (24, 25). ANCOVA further confirmed that dimerization domain and DNA-binding domain mutation were independently associated with lower FCP levels. A comparison of the activity of the mutant and wild-type proteins revealed that mutations in the transactivation domain disrupted transcriptional activity less than mutations in the DNA-binding domain, as it primarily affected coactivator recruitment rather than DNA-binding affinity (26). This study is the first to reveal in a Chinese population that dimerization domain and DNA-binding domain mutations in HNF1A-MODY are associated with poorer pancreatic islet function. This finding provides a basis for assessing patient prognosis and formulating treatment strategies.

Regarding treatment, sulfonylureas are widely acknowledged as the first-line therapy for HNF1A-MODY (23); however, a notable proportion of patients in our study were treated with insulin. Real-world data and reviews indicate that insulin use is indeed common among genetically confirmed HNF1A-MODY patients. The real-world study (27) revealed that the insulin usage rate within one year after diabetes diagnosis was 52% (35% insulin monotherapy, 17% insulin combined with oral hypoglycemic agents). Even after genetic diagnosis of HNF1A-MODY, insulin usage remained at 42% (25% insulin alone, 17% insulin combined with oral agents). A review on the clinical characteristics of HNF1A-MODY patients (10) reported that insulin usage was 16.7% in those with dimerization domain mutations, 33.8% in DNA-binding domain, and 33.2% in transactivation domain. In our study, after excluding patients with variants of uncertain significance, the corresponding insulin usage rates for these domains were 33.3%, 35.3%, and 45.28%, respectively. The widespread use of insulin in HNF1A-MODY is likely attributable to diagnostic delay or treatment patterns, rather than true insulin dependence. Therefore, inferring domain-specific treatment responses based on current insulin use should be approached with caution. Instead, prospective therapeutic trials with sulfonylurea should be systematically conducted following genetic diagnosis.

In addition, our data showed that patients with DNA-binding domain mutations had lower LDL-C and TC levels, but higher HDL-C levels. HNF1α is involved in bile acid and plasma cholesterol metabolism (28). Prior studies have shown that liver-specific knockdown of HNF1α reduces LDL-C levels in normolipidemic mice (29), though similar effects have not been observed in humans. Our results suggest a potential domain-specific correlation with lipid regulation, but the precise mechanisms require further investigation.

This study also characterized the clinical profile and short-term treatment patterns associated with different glycemic control statuses. After a 2-year follow-up of the patients from Qilu Hospital, the majority achieved target glycemic levels; however, some developed new-onset diabetic peripheral neuropathy or diabetic retinopathy within this observation period. Notably, poor glycemic control was associated with higher baseline levels of BMI and blood lipids. These findings appear to support the association between chronic hyperglycemia and microvascular complications in this cohort, and may also underscore the potential role of weight and lipid management in short-term metabolic outcomes. Regarding pharmacotherapy, patients with good glycemic control were more likely to use sulfonylurea monotherapy, whereas those with poor control required insulin-based combination therapy. This observation aligns with the characteristic hypersensitivity to sulfonylureas in HNF1A-MODY patients, for whom these agents are considered first-line therapy (23). Although recent case reports identified novel *HNF1A* variants exhibiting resistance to sulfonylureas and other glucose-lowering drugs (9, 30), such variants were not identified in our cohort. Given that HNF1A-MODY patients exhibit progressive β-cell dysfunction and remain at high risk for both microvascular and macrovascular complications (31–33), early diagnosis coupled with stringent glycemic control is crucial for reducing the prevalence of late-diabetic complications (34, 35). However, given the relatively short follow-up period, longer-term studies will be needed to clarify prognosis and the durability of treatment effects.

This study has several limitations. First, the relatively small sample size, particularly the low number of cases in the dimerization domain group may affect statistical power and limit the generalizability of the findings. Second, the relatively short follow-up period is insufficient for comprehensively assessing long-term complication risks and does not provide strong support for long-term prognosis. To evaluate the impact on long-term outcomes, we are conducting ongoing follow-up of the patient cohort. Third, literature-derived cases exhibited variability in diagnostic criteria and laboratory methods, along with incomplete clinical variables, which may introduce selection and measurement biases and increase clinical data heterogeneity. Fourth, while we performed a sensitivity analysis excluding patients with early insulin dependence, the overall high prevalence of insulin therapy in our combined cohort may still reflect regional diagnostic delays or historical treatment patterns rather than intrinsic sulfonylurea unresponsiveness. This could introduce residual confounding when assessing metabolic phenotypes. Moreover, a subset of patients in the adolescent type 2 diabetes control group did not undergo genetic testing, which may to some extent affect the specificity of intergroup comparisons. Furthermore, the absence of functional studies on the identified variants restricts mechanistic interpretation of domain−specific mutation effects. Future multicenter studies with larger cohorts and longer follow-up durations, combined with functional characterization of mutant proteins, are needed to better elucidate the predictive value of different variant types for drug response and complication risks.

## 5 Conclusion

In conclusion, this study demonstrated domain-specific effects of *HNF1A* mutations. Pancreatic islet function was significantly associated with the location of the mutation. Mutations in the dimerization domain and DNA-binding domains were associated with an earlier age of onset and more severe insulin secretion defects. Regarding lipid metabolism, our analyses indicated an observational association between DNA-binding domain mutations and a distinct lipid profile characterized by lower LDL-C and higher HDL-C levels. This study summarized the association between clinical phenotypes and genotypes in patients with HNF1A-MODY, revealed the correlation between *HNF1A* mutation regions and pancreatic islet function as well as blood lipid levels, thereby providing a theoretical foundation for predicting pancreatic islet function prognosis through gene sequencing and guiding individualized treatment options.

## Data Availability

The original contributions presented in the study are included in the article/**Supplementary Material**. Further inquiries can be directed to the corresponding authors.
