# Supplementary material for: Genotype-phenotype correlations of fasting C-peptide and lipids in HNF1A-MODY: a single-center series and multi-center cross-sectional analysis in Chinese population
Source: Front Endocrinol (Lausanne). 2026 Feb 12;17:1735596. doi: 10.3389/fendo.2026.1735596 (PMC12935643; doi:10.3389/fendo.2026.1735596)
Supplement: Supplementary file 1 [file DataSheet1.docx]

Supplementary Material

# Appendix S1: Search strategy

#1 "maturity onset diabetes of the young, type 3"[Supplementary Concept]

#2 (((((Maturity-Onset Diabetes of the Young, Type 3[Title/Abstract]) OR (MODY3[Title/Abstract])) OR (HNF1A-MODY[Title/Abstract])) OR (HNF1α-MODY[Title/Abstract])) OR (HNF1-alpha MODY[Title/Abstract])) OR (MODY, type 3[Title/Abstract])

#3 #1 OR #2

#4 (hnf1a protein human[Supplementary Concept]) OR (Hepatocyte Nuclear Factor 1-alpha[MeSH Terms])

#5 ((((Hepatocyte Nuclear Factor 1-alpha[Title/Abstract]) OR (HNF1A[Title/Abstract])) OR (HNF1α[Title/Abstract])) OR (HNF1 homeobox A protein[Title/Abstract])) OR (HNF1-alpha[Title/Abstract])

#6 #4 OR #5

#7 Diabetes Mellitus[MeSH Terms]

#8 diabetes[Title/Abstract]

#9 #7 OR #8

#10 #6 AND #9

#11 #3 OR #10

#12 (((((((((China[Affiliation]) OR (China[Title/Abstract])) OR (Chinese[Title/Abstract])) OR (Chinese[Affiliation])) OR (Taiwan[Affiliation])) OR (Taiwan[Title/Abstract])) OR (Hong Kong[Title/Abstract])) OR (Hong Kong[Affiliation])) OR (Macau[Affiliation])) OR (Macau[Title/Abstract])

#13 #11 AND #12

# Supplementary Tables

**Supplementary Table 1**

| **Article** |  | **Sex** | **Mutation site** | **Age of onset (years)** | **BMI (kg/m^2^)** | **Family history** | **FPG (mmol/L)** | **2hPG (mmol/L)** | **FCP (ng/ml)** | **2hPCP(ng/ml)** | **Islet autoantibody** | **HbA_1c_ (%)** | **TG (mmol/L)** | **TC (mmol/L)** | **HDL-C (mmol/L)** | **LDL-C (mmol/L)** | **Complications** | **Treatment** |
| --- | --- | --- | --- | --- | --- | --- | --- | --- | --- | --- | --- | --- | --- | --- | --- | --- | --- | --- |
| (1) | 1 | F | c.802T>A, p.Phe268Ile | 8.42 | 23.02 | yes | 7.20 |  | 2.19 |  | - | 13.40 |  |  |  |  | none | Sulfonylureas |
|  | 2 | M | c.1502-2A>G | 10.75 | 17.48 | yes | 5.60 |  | 1.08 |  | - | 7.32 |  |  |  |  | none | Sulfonylureas |
|  | 3 | F | c.34C>T, p.Leu12Phe | 12.42 | 22.5 | yes | 6.22 |  | 1.11 |  | - | 8.40 |  |  |  |  | none | Sulfonylureas |
|  | 4 | F | c.526+1G>A | 17.83 | 24.43 | yes | 6.00 |  | 1.08 |  | - | 6.80 |  |  |  |  | none | Sulfonylureas |
|  | 5 | F | c.1375_1379delinsTTGC, p.Gln460Argfs*25 | 10.33 | 20.3 | yes | 5.10 |  | 1.26 |  | IAA(＋) | 8.30 |  |  |  |  | none | Metformin+INS |
| (2) | 6 | F | c.47T>A, p.Leu16Gln | 23 | 17.3 | 6.38 | 19.30 | 6.38 | 1.16 | 3.19 | - | 6.50 | 0.74 | 5.01 |  | 2.90 | none | AGI+INS |
|  | 7 | M |  | 19 | 18.4 | 3.85 | 12.18 | 3.85 | 1.12 | 11.87 | - | 6.20 |  |  |  |  | none | Lifestyles |
| (3) | 8 | M | c.1508_1509delAC, p.Tyr503Ter | 35 | 19.81 | 6.67 | 11.53 | 6.67 | 1.03 | 5.96 | - | 6.80 |  |  |  |  | DKD | Metformin |
| (4) | 9 | F | c.865dup, p.Gly292fs | 13 | 21.87 | 17.19 | 30.00 | 17.19 | 1.90 | 3.58 | - | 14.40 |  |  |  |  | none | AGI+INS |
| (5) | 10 | F | c.G812A, p.R271Q | 19 | 23.1 | 3.32 | 16.47 | 3.32 | 0.05 | 3.34 | - | 9.60 |  |  |  |  | DPN | Sulfonylureas |
| (6) | 11 | M | c.G392T, p.R131L | 12 | 17.7 | 7.40 | 18.50 | 7.40 | 1.32 | 2.76 | - | 9.00 | 0.83 | 5.08 | 1.14 | 2.61 | none | Glimepiride |
| (7) | 12 | F | c.779C＞T, p.T260M | 10 | 18 | 8.10 | 25.30 | 8.10 | 1.50 | 2.40 | - | 10.50 |  |  |  |  | none | Gliclazide |
| (8) | 13 | F | c.814C＞A, p.R272S | 13 | 21.8 | yes | 16.82 | 28.39 | 1.10 | 2.71 | - | 10.80 | 1.94 | 4.32 | 1.48 | 2.34 | none | INS |
|  | 14 | F |  | 23 | 24.19 | no | 8.00 | 13.10 |  |  | - | 7.10 | 1.87 | 3.79 | 1.59 | 2.32 | DKD | Glibenclamide+Metformin |
|  | 15 | F |  | 14 | 23.24 | no | 9.50 | 14.70 |  |  | - | 8.20 | 2.14 | 5.39 | 2.75 | 4.54 | DKD、DR | Sulfonylureas |
|  | 16 | F |  | 11 | 24.3 | no | 6.50 | 10.90 |  |  | - | 6.90 | 1.45 | 2.48 | 2.63 | 3.87 | none | Glimepiride+Metformin |
|  | 17 | M |  | 16 | 22.7 | yes | 7.80 | 13.40 |  |  | - | 8.90 | 1.39 | 4.17 | 1.28 | 2.43 | DR | INS |
|  | 18 | M |  | 15 | 19.6 | yes | 4.90 | 8.90 |  |  | - | 6.20 | 0.79 | 2.60 | 0.85 | 1.86 | none | Lifestyles |
|  | 19 | F |  | 16 | 25.1 | yes | 16.82 | 23.61 |  |  | - | 10.10 | 1.43 | 3.10 | 1.35 | 3.38 | none | Glimepiride |
| (9) | 20 | F | c.293C>T, Ala98Val | 14 | 16.3 | yes | 6.20 | 6.80 |  |  | - | 5.70 | 1.65 | 3.57 |  |  | none | Sulfonylureas |
| (10) | 21 | F | c.1136_1137delCT, p.Pro379ArgfsX39 | 14 | 20.69 | yes | 14.21 |  |  |  | - |  |  |  |  | 3.23 | none | INS |
| (11) | 22 | M | c.1333_1334del, p.S445Cfs*103 | 13 | 22.99 | yes | 5.16 | 14.11 | 2.62 | 8.59 | - | 7.50 |  |  |  |  | none | Glimepiride |
| (12) | 23 | M | c.1135C>A, p.Pro379Thr | 15 | 21.72 | yes | 7.07 | 21.52 | 0.72 | 1.46 | - | 13.80 |  |  |  |  | none | Repaglinide+DPP-4i+AGI+INS |
| (13) | 24 | F | c.C787T, p.R263C | 10 | 23.9 | yes | 7.20 | 24.00 | 1.77 | 3.05 | - | 7.10 | 0.94 | 4.53 | 1.05 | 2.92 | DR | Metformin+DPP-4i+SGLT-2i+INS |
| (14) | 25 | F | c.872dup, p.G292Rfs*25 | 12.4 | 24.53 | yes | 12.60 |  | 1.00 |  | - | 8.90 |  |  |  |  | none | Glimepiride |
| (15) | 26 | M | c.977C>T, p.A326V | 31 | 19.03 | no | 8.48 | 20.18 | 0.30 | 0.60 | - | 11.46 |  |  |  |  | DKD | INS |
| (16) | 27 | F | c.C1135A, p.P379T | 18 | 20.12 | yes | 7.55 | 15.50 | 2.40 | 7.09 | - | 9.60 | 1.79 | 5.05 |  |  | none | OHA |
|  | 28 | M | c.557_560delTTGA, p.I186Kfs*46 | 16 | 19.89 | no | 8.60 | 11.10 | 0.58 | 1.25 | - | 8.80 | 1.78 | 4.23 |  |  | none | INS |
| (17) | 29 | M | c.1854C>G, p.I618M | 11 | 17.2 | no | 17.98 | 11.94 | 1.52 | 2.15 | - | 17.40 | 2.59 | 5.65 | 1.15 | 3.62 | none | Glimepiride+INS |
| (18) | 30 | F | c.1137delT, p.Val379fs | 24 | 22.7 | yes | 5.80 | 12.60 | 1.45 | 4.18 | - | 6.80 |  | 7.18 |  |  | Macrovascular disease、DPN、DR、DKD | Gliquidone+DPP-4i+INS |
| (19) | 31 | M | c.872dupC, p.Gly292fs | 39 | 22.85 | yes | 5.66 | 12.18 | 0.69 | 1.08 | - | 9.30 |  |  |  |  | DR | Repaglinide |
| (20) | 32 | F | c.865dupC, p.G292Rfs*25 | 26 | 20.47 | no | 9.00 | 17.40 | 0.63 | 2.13 | - | 7.50 | 1.15 |  | 1.01 | 2.55 | DPN、DR、DKD | INS+DPP-4i |
|  | 33 | F | c.961C>A, p.R321S | 21 | 26.31 | yes | 8.72 | 14.00 | 2.23 | 8.10 | - | 5.40 | 4.24 |  | 0.88 | 2.09 | none | Gliclazide+Metformin |
|  | 34 | F | c.323T>C, p.L108P | 12 | 19.74 | yes | 10.73 |  | 1.96 | 3.21 | - | 12.10 | 1.28 |  | 0.92 | 1.34 | DPN | Gliclazide |
| (21) | 35 | F | p.P379T | 22 | 22.86 | yes | 5.90 | 19.00 | 2.32 | 5.92 | - | 7.50 | 0.80 | 3.84 | 0.93 | 2.59 | none | Gliclazide |
| (22) | 36 | M | c.779C>T, p.Thr260Met | 12 | 20.8 | yes | 8.20 | 17.30 |  |  | - | 9.30 |  |  | 1.49 |  | none | Repaglinide |
| (23) | 37 | M | c.598C>T, p.Arg200Trp | 25 | 20.24 | yes | 6.6 | 13.6 | 0.7 | 1.04 | - | 7.53 | 4.55 |  | 1.35 |  | DPN、DR | Sulfonylureas+INS |
| (24) | 38 | F | c.511C>T, p.Arg171* | 12 | 21.11 | yes | 7.85 | 12.11 | 1.07 | 2.61 | - | 7.8 |  |  |  |  | none | INS |
|  | 39 | M | c.511C>T, p.Arg171* | 35 | 22.64 | yes | 9.51 | 13.25 | 1.01 | 2.54 | - | 9.1 |  |  |  |  | none | Metformin |
| (25) | 40 | F | c.C1531T, p.Q511X | 16 | 19.31 | yes | 8.7 | 15.1 | 0.69 | 0.85 | - | 15.7 | 0.49 | 4.93 | 1.86 | 2.77 | none | Gliclazide |
|  | 41 | F | c.C1531T, p.Q511X | 23 | 20.81 | yes | 7.14 | 10 | 0.75 | 1.81 | - | 6.5 | 1.5 | 7.66 | 1.58 | 5.37 | DKD | INS |
| (26) | 42 | F | c.3G>A, p.M1I | 17 | 23.37 | yes | 3.76 | 6.88 | 1.19 | 3.14 | - | 6.30 |  |  |  |  | none | Metformin |
|  | 43 | M | c.671C>A, p.P224H | 13 | 26.77 | yes | 12.91 | 16.32 | 1.00 | 1.51 | - | 10.80 |  |  |  |  | none | Gliclazide+Pioglitazone |
|  | 44 | M | c.671C>A, p.P224H | 12 | 25.91 | yes | 11.36 | 17.07 | 1.30 | 2.04 | - | 9.00 |  |  |  |  | none | INS |
|  | 45 | M | c.671C>A, p.P224H | 30 |  | yes | 7.71 | 20.93 | 0.43 | 1.57 | - | 8 |  |  |  |  | none | INS |
| (27) | 46 | F | c.1623+29C>T | 31 | 22.9 | yes | 5.92 |  | 1.3 |  | - | 7.1 |  |  |  |  | DPN、DR、DKD | Metformin+AGI+INS |
|  | 47 | F | c.1623+29C>T | 18 | 22.7 | yes | 5.42 | 12.35 | 2.17 |  | - | 5.6 |  |  |  |  | none | Lifestyles |
|  | 48 | M | c.1623+29C>T | 15 | 23.8 | yes | 5.8 | 10.3 | 2.58 |  | - | 5.8 |  |  |  |  | none | Lifestyles |
|  | 49 | M | c.1623+29C>T | 15 | 24.6 | yes | 6.7 | 10.93 | 2.41 |  | - | 6.9 |  |  |  |  | none | Metformin |
| (28) | 50 | M | c.874dupA, p.G292Rfs*25 | 21 | 18.11 | yes | 5.3 | 7.4 | 1.94 | 4.41 | - | 5.9 |  |  |  |  | none | Lifestyles |
|  | 51 | M | c.874dupA, p.G292Rfs*25 | 38 | 20.24 | yes | 8 | 12.5 | 1.08 | 2.59 | - | 6.5 |  |  |  |  | none | Sulfonylureas+INS |
| (29) | 52 | M | c.79A＞C, p.Ile27Leu | 20 | 33.95 | yes | 7.4 | 21 | 1.43 | 4.28 | - | 7.4 | 3.54 | 5.32 | 0.98 | 2.56 | none | OHA+INS |
|  | 53 | F | c.1720G＞A, p.Gly574Ser | 24 | 26.71 | yes | 8.52 | 25.9 | 0.76 | 2.96 | - | 12.7 | 1.75 | 5.68 | 1.18 | 4.33 | Macrovascular disease、DPN | OHA+INS |
|  | 54 | M | c.1460G＞A, p.Ser487Asn | 25 | 30.46 | yes | 9.8 | 22.6 | 4.3 | 9.34 | - | 10.02 | 4.89 | 5.07 | 0.71 | 3.08 | Macrovascular disease、DPN、DR、DKD | OHA+INS |
|  | 55 | M | c.79A＞C, p.Ile27Leu | 23 | 23.71 | yes | 8.5 | 19.4 | 0.45 | 1.49 | - | 12.4 | 3.23 | 5.69 | 0.86 | 3.53 | DPN | INS |
|  | 56 | M | c.79A＞C, p.Ile27Leu | 13 | 17.1 | yes | 16.3 | 20.5 | 0.61 | 1.5 | - | 19.74 | 1.37 | 4.5 | 0.90 | 3.36 | none | INS |
| (30) | 57 | F | c.P112L, p.Pro112Leu | 14 | 20.28 | yes | 8.20 | 25.00 | 0.83 | 1.25 | - | 12.10 | 5.06 | 6.57 | 2.86 |  | none | Metformin+DPP-4i+INS |
| (31) | 58 | F | c.335delC, p.P112Rfs*43 | 15 | 20.12 | yes |  |  |  |  | - |  |  |  |  |  | DR | SGLT-2i+INS |
| (32) | 59 | F | c.335delC, p.P112Rfs*43 | 26 | 19 | yes |  |  | 0.54 |  | - | 13.00 |  |  |  |  | DKD、DR | Glimepiride |
| (33) | 60 | M | c.1330_1331del, p.Gln444fs | 9 | 15.56 | yes | 13 | 18 | 0.4 | 0.5 | - | 15.50 | 1.4 | 3.32 | 0.88 | 1.92 | none | Metformin+Gliclazide+AGI+INS |
| (34) | 61 | M | c.2T>C, p.Met1? | 23 | 18.1 | yes | 6.82 | 11.12 | 1.14 | 2.91 | - | 6.80 | 1.59 | 4.1 | 1.59 | 2.48 | none | Gliclazide |
|  | 62 | F | c.1136_1137del, p.Pro379fs | 12 | 16.7 | yes | 8.98 | 20.37 | 0.69 | 0.93 | - | 14.40 | 1.64 | 4.98 | 1.44 | 2.76 | DPN、DKD | Gliclazide+INS |
| (35) | 63 | F | c.788G>A, p.R263H | 11 | 19.4 | yes | 8.2 | 16.2 | 1.35 | 2.3 | - | 7.80 | 0.77 |  |  |  | none | Glimepiride |
| (36) | 64 | M | c.C29T, p.T10M | 39 | 21.95 | no | 10.6 |  | 1.78 | 4.92 | - | 8.10 |  |  |  |  | none | Lifestyles |
|  | 65 | F | c.C29T, p.T10M | 41 | 27.59 | yes | 8.73 |  | 1.08 | 1.96 | - | 7.60 |  |  |  |  | none | OHA |
|  | 66 | M | c.C29T, p.T10M | 36 | 21.45 | no | 10.53 |  | 0.74 | 0.91 | - | 9.80 |  |  |  |  | none | Lifestyles |
|  | 67 | M | c.C1135A, p.P379T | 42 | 24.09 | no | 8.4 |  | 2.26 | 5.86 | - | 8.50 |  |  |  |  | none | INS |
|  | 68 | M | c.1624-2A > T | 32.3 |  | yes | 6.11 |  | 2.77 | 12.76 | - | 5.30 |  |  |  |  | none | OHA |
|  | 69 | F | c.1624-2A > T | 43 | 28.62 | yes | 13.35 |  | 1.31 | 4.79 | - | 8.40 |  |  |  |  | none | Lifestyles |
| (37) | 70 | F | c.512G>A, p.Arg171Gln | 34 | 20.83 | yes | 5.2 | 13.6 | 2.09 | 3.85 | - | 6.5 | 0.42 | 4.08 | 1.6 | 2.29 | none | Glimepiride |
|  | 71 | M | c.391C>T, p.Arg131Trp | 24 | 21.98 | yes | 10 |  | 1 |  | - | 10 | 2.2 | 5.7 | 1 | 2.8 | DKD、DR | Glimepiride+INS |
|  | 72 | F | c.391C>T, p.Arg131Trp | 17 | 19.53 | yes | 8.5 | 20.4 | 1.75 | 4.04 | - | 10.2 | 1.15 | 3.98 | 1.28 | 2.98 | none | Glimepiride |
| (38) | 73 | M | c.598C>T, p.Arg200Trp | 25 | 20.24 | yes | 7.04 | 9.42 | 0.70 | 1.04 | - | 7.53 | 4.55 |  | 1.35 |  | DPN | DPP-4i+INS |
| (39) | 74 | F | c.1502+6G>A | 15 | 23 | yes | 10.5 | 19.96 |  |  | - | 10 | 1.04 | 5.66 | 1.03 | 4.16 | none | Gliquidone |
|  | 75 | F | IVS7-6G > A | 30 | 27 | yes | 5.81 | 18.78 |  |  | - |  | 2.91 | 8.11 | 1.21 | 5.05 | Macrovascular disease、DPN、DR、DKD | SGLT-2i+INS |
| (40) | 76 | M | c.1108-1G>A | 22 | 24.7 | yes | 7.48 | 15.94 | 2.89 | 4.65 | - | 7.2 | 0.78 |  | 1.07 | 1.95 | none | Gliclazide |
| (41) | 77 | F | Arg263Cys | 14 | 22.8 | yes | 6.04 |  |  |  | - | 16 | 0.88 | 4.24 | 1.32 | 2.59 | none | OHA+INS |
|  | 78 | F | Glu240Val | 13 | 20.7 | yes | 4.19 |  |  |  | - | 5.4 | 0.43 | 4.05 | 1.75 | 1.66 | DR | INS |
|  | 79 | M | Arg131Trp | 33 | 18 | yes | 7.28 |  |  |  | - | 7.5 | 1.05 | 3.98 | 1.35 | 2.33 | none | OHA |
|  | 80 | F | Arg200Trp | 11 | 21.9 | yes | 5.43 |  |  |  | - | 9.4 | 1.01 | 4.49 | 1.39 | 2.65 | none | OHA |
|  | 81 | F | Arg200Trp | 18 | 20.8 | yes | 6.44 |  |  |  | - | 6.1 | 0.86 | 3.12 | 1.69 | 2.72 | DKD、DR | OHA |
|  | 82 | F | Ivs8-1G>A | 13 | 22.7 | yes | 8.96 |  |  |  | - | 7.7 | 1.18 | 4.28 | 1.5 | 2.55 | none | INS |
|  | 83 | M | Arg159fs | 32 | 21.6 | no | 7.62 |  |  |  | - | 7.7 | 1.54 | 6.15 | 1.25 | 3.88 | DKD、DR | OHA |
|  | 84 | F | Gln324Ter | 22 | 21.8 | yes | 9.57 |  |  |  | - | 7.6 | 1.88 | 7.51 | 1.53 | 4.7 | DKD | INS |
|  | 85 | M | Pro112Leu | 29 | 17.5 | yes | 8.12 |  |  |  | - | 6.9 | 1.51 | 4.26 | 1.26 | 2.52 | none | OHA |
| (42) | 86 | M | c. 686G>A, p. Arg229Gln | 26 | 22.2 | yes | 7.5 | 9.1 | 0.83 | 4.3 | - | 5.8 | 0.8 | 4.04 | 1.14 | 2.28 | none | Sulfonylureas |
|  | 87 | M | c.526 + 1G>A | 13 | 20.2 | yes | 4.9 | 11.3 | 0.72 | 3.75 | - | 5.7 | 0.54 | 3.7 | 1.14 | 2.19 | none | Lifestyles |
| (43) | 88 | M | c.779 C>T, p.T260M | 21 | 19.96 | yes | 8.32 | 20.02 | 1.02 | 2.78 | - | 7.1 | 1.24 | 4.98 |  |  | none | Gliclazide |
|  | 89 | F | c.779 C>T, p.T260M | 18 | 16.45 | yes | 6.09 | 11.65 | 0.68 | 1.57 | - |  | 1.06 | 5.49 | 1.57 | 3.53 | none | Glimepiride |
| (44) | 90 | F | c.1130_1131insC, p.V380Cfs*39 | 23 | 18.66 | yes | 7.76 | 19.84 | 0.70 | 2.44 | ICA,IAA(＋) | 5.40 | 0.77 | 4.00 | 1.20 | 2.45 | DPN、DR、DKD | INS |
|  | 91 | F | c.1130_1131insC, p.V380Cfs*39 | 24 | 17.3 | yes | 10.88 |  | 0.92 | 1.80 | - | 9.20 | 0.84 | 4.40 | 0.99 | 3.03 | DPN、DR | Gliclazide |
|  | 92 | F | c.1130_1131insC, p.V380Cfs*39 | 36 | 19.6 | yes | 6.11 | 10.12 | 0.92 | 3.61 | - | 6.30 | 1.11 | 2.60 | 0.47 | 1.63 | DPN、DR | Lifestyles |
|  | 93 | F | c.1130_1131insC, p.V380Cfs*39 | 11 | 19.6 | yes | 5.88 | 13.37 | 1.59 | 3.75 | - | 7.3 | 0.85 | 4.15 | 1.52 | 2.24 | none | Lifestyles |
| (45) | 94 | F | R54X | 19 | 22 | yes | 18 | 21.56 | 1.48 |  | - | 15 |  |  |  |  | none | Glibenclamide |
|  | 95 | M | Pro379Ala | 35 | 24.7 | yes | 6.8 | 13.2 |  |  | - | 8 | 3.52 | 6 | 1.6 | 3.7 | none | INS |
|  | 96 | F | Pro379Ala | 36 | 24.4 | yes | 8.2 | 11 |  |  | - | 8 | 2.25 | 5 | 1.3 | 3.1 | none | INS |
|  | 97 | F | Pro379Ala | 39 | 28.5 | yes | 6.00 | 5.80 |  |  | - | 5.50 | 2.19 | 5.00 | 1.50 | 3.10 | none | Lifestyles |
| (46) | 98 | F | IVS2nt-1G>A | 19 | 19.6 | yes |  |  | 0.47 |  | - | 8 |  |  |  |  | DKD、DR | INS |
|  | 99 | F | IVS2nt-1G>A | 24 | 22.7 | yes |  |  | 0.86 |  | - | 6.4 |  |  |  |  | DKD、DR | INS |
|  | 100 | F | IVS2nt-1G>A | 33 | 18.5 | yes |  |  | 0.32 |  | - | 17.2 |  |  |  |  | Macrovascular disease、DPN、DR、DKD | INS |
|  | 101 | F | IVS2nt-1G>A | 12 | 18.8 | yes |  |  | 0.13 |  | - | 8.8 |  |  |  |  | none | INS |
|  | 102 | M | IVS2nt-1G>A | 15 | 16.6 | yes |  |  | 0.36 |  | - | 5.3 |  |  |  |  | none | INS |
| (47) | 103 | M | c.391 C>T, p.R131W | 11 | 17.55 | yes | 6.1 |  | 1.36 |  | - | 8.9 |  |  |  |  | none | Sulfonylureas |
|  | 104 | F | c.757 G>A, p.G253R | 15.4 | 21.8 | yes | 5.7 |  | 1.93 |  | - | 5.7 |  |  |  |  | none | Sulfonylureas |
|  | 105 | F | c.758 G>A, p.G253E | 9.5 | 19.6 | yes | 6.7 |  | 1.7 |  | - | 7.4 |  |  |  |  | none | Sulfonylureas |
|  | 106 | F | c.779 C>T, p.T260M | 9.8 | 16.2 | yes | 7.1 |  | 2.34 |  | - | 6.6 |  |  |  |  | none | Sulfonylureas |
|  | 107 | F | c.1576 G>A, p.D526N | 10 | 18.4 | yes | 6.3 |  | 1.82 |  | - | 6.3 |  |  |  |  | none | Sulfonylureas |
| (48) | 108 | M | G→A, p.G20R | 16 | 20 | yes | 4.9 |  | 0.48 |  |  |  |  |  |  |  | none | Lifestyles |
|  | 109 | M | C→G, p.I618M | 38 | 29 | yes | 6.6 |  | 0.3 |  |  |  |  |  |  |  | none | OHA |
|  | 110 | F | IVS2nt-1G→A | 19 | 26 | yes | 13.9 |  | 1.41 |  |  | 8.7 |  |  |  |  | DPN、DR、DKD | INS |
|  | 111 | F | G→A, p.R203H | 33 | 20 | yes | 4.9 |  | 0.33 |  |  | 6 |  |  |  |  | none | OHA |
|  | 112 | M | C→G, p.S432C | 30 | 23 | yes | 7.4 |  | 1.47 |  |  | 7.1 |  |  |  |  | none | OHA |
| (49) | 113 | M | A→G, Y218C | 23 | 19.81 | yes | 22 |  | 0.69 | 2.04 |  | 7.4 | 1.02 | 4.19 |  |  | DR | Gliclazide+Metformin |

Abbreviations: BMI, body mass index; FPG, fasting plasma glucose; 2hPG, 2-hour postprandial blood glucose; FCP, fasting C-peptide; 2hPCP, 2-hour postprandial C-peptide; HbA1c, glycated hemoglobin; TG, triglyceride; TC, total cholesterol; HDL-C, high-density lipoprotein cholesterol; LDL-C, low-density lipoprotein cholesterol; DKD, diabetic kidney disease; DR, diabetic retinopathy; NPDR, non-proliferative diabetic retinopathy; DPN, diabetic peripheral neuropathy; DPP-4i, dipeptidyl peptidase 4 inhibitor; AGI, alpha glucosidase inhibitor; INS, insulin; SGLT-2i, sodium-glucose transporter 2 inhibitor; OHA, oral hypoglycemic drugs.

**Supplementary Table 2** Clinical characteristics of patients with HNF1A-MODY by different domains in Qilu Hospital.

|  | **Total (n=17)** | **Dimerization domain (n=2)** | **DNA-binding domain (n=8)** | **Transactivation domain (n=7)** | ***p*-value** |
| --- | --- | --- | --- | --- | --- |
| Age of onset (years) | 15.00 (11.00, 23.00) | 16.50 (16.00, 17.00) | 13.50 (10.25, 24.00) | 12.00(11.00, 25.00) | 0.783 |
| Age of diagnosis (years) | 18.18±6.59 | 16.50 (16.00, 17.00) | 18.38±7.54 | 18.43±6.95 | 0.937 |
| Classic diabetes symptoms (%) | 9/17 (52.0) | 1/2 (50.0) | 6/8 (75.0) | 2/7 (28.6) | 0.218 |
| BMI (kg/m^2^) | 20.95±3.08 | 19.34 (17.31, 21.36) | 20.21±2.04 | 22.48±4.04 | 0.308 |
| Family history (%) | 15/17 (88.2) | 1/2 (50.0) | 8/8 (100.0) | 6/7 (85.7) | 0.110 |
| FPG (mmol/L) | 8.96±4.67 | 10.19 (5.43, 14.94) | 7.90±3.04 | 9.82±6.08 | 0.703 |
| 2hPG (mmol/L) | 13.14 (11.02, 20.48) | 16.12 (12.73, 19.50) | 15.22 (11.97, 23.51) | 11.25 (10.42, 19.52) | 0.645 |
| FCP (ng/ml) | 1.11±0.59 | 0.63 (0.41, 0.85) | 1.07±0.34 | 1.31±0.81 | 0.370 |
| 2hPCP (ng/ml) | 3.17±1.54 | 2.33 (1.08, 3.57) | 2.85±1.64 | 3.87±1.36 | 0.415 |
| Islet autoantibody | 1/17 (5.9) | 0 | 1/8 (12.5) | 0 | 1.000 |
| HbA_1c_ (%) | 9.31±2.92 | 13.15 (8.50, 17.80) | 9.19±1.99 | 8.36±2.21 | 0.117 |
| HOMA-IR | 3.22±2.03 | 3.44 | 3.67±2.94 | 2.81±1.52 | 0.848 |
| TyG | 8.59±0.69 | 8.69 (8.44, 8.93) | 8.41±0.42 | 8.78±0.97 | 0.589 |
| TyG-BMI | 181.86±36.51 | 168.48 (146.16, 190.80) | 169.87±19.43 | 202.31±49.68 | 0.233 |
| HOMA-β (ins) | 104.11±72.67 | 147.67 | 132.99±96.94 | 72.30±47.55 | 0.426 |
| HOMA-β (CP) | 2.75±2.42 | 1.59 (0.24, 2.93) | 2.91±2.61 | 2.90±2.57 | 0.794 |
| TG (mmol/L) | 0.78 (0.65, 0.89) | 1.07 (0.39, 1.75) | 0.80 (0.74, 0.87) | 0.75 (0.65, 1.02) | 0.944 |
| TC (mmol/L) | 4.11 (3.39, 4.46) | 5.17 (4.25, 6.08) | 4.10 (2.66, 4.12) | 3.89 (3.31, 4.80) | 0.160 |
| HDL-C (mmol/L) | 1.36±0.28 | 1.65 (1.26, 2.04) | 1.37±0.26 | 1.26±0.19 | 0.228 |
| LDL-C (mmol/L) | 2.23±0.76 | 2.95 (2.51, 3.39) | 1.84±0.61 | 2.46±0.78 | 0.095 |
| UA (umol/L) | 316.40±50.67 | 308 | 295.00±10.98 | 345.25±75.13 | 0.372 |
| Diabetic kidney disease (%) | 0 | 0 | 0 | 0 |  |
| Diabetic peripheral neuropathy (%) | 5/16 (31.3) | 0 | 3/8 (37.5) | 2/6 (33.3) | 0.808 |
| Diabetic retinopathy (%) | 4/16 (25.0) | 0 | 3/8 (37.5) | 1/6 (16.7) | 0.769 |
| Microvascular complications (%) | 0 | 0 | 0 | 0 |  |
| Lifestyles (%) | 2/27 (11.8) | 0 | 0 | 2/7 (28.6) | 0.382 |
| OHA (%) | 10/17 (58.8) | 1/2 (50.0) | 6/8 (75.0) | 3/7 (42.9) | 0.521 |
| INS (%) | 1/17 (5.9) | 0 | 1/8 (12.5) | 0 | 1.000 |
| OHA+INS (%) | 4/17 (23.5) | 1/2 (50.0) | 1/8 (12.5) | 2/7 (28.6) | 0.424 |
| lipid-lowering therapy | 0 | 0 | 0 | 0 |  |

Abbreviations: Classic diabetes symptoms, polydipsia, polyuria, polyphagia, weight loss; BMI, body mass index; FPG, fasting plasma glucose; 2hPG, 2-hour postprandial blood glucose; FCP, fasting C-peptide; 2hPCP, 2-hour postprandial C-peptide; HbA1c, glycated hemoglobin; HOMA-IR, homeostasis model assessment of insulin resistance; TyG, triglyceride-glucose; TyG-BMI, triglyceride-glucose-body mass index; HOMA-β (ins), homeostasis model assessment β cell function (based on Insulin); HOMA-β (CP), homeostasis model assessment β cell function (based on C-Peptide); TG, triglyceride; TC, total cholesterol; HDL-C, high-density lipoprotein cholesterol; LDL-C, low-density lipoprotein cholesterol; UA, uric acid; OHA, oral hypoglycemic drugs; INS, insulin; a: Compared with the dimerization domain, *p* < 0.05; b: Compared with the DNA-binding domain, *p* < 0.05; *: *p* < 0.05.

**Supplementary Table 3**

Analysis of covariance of LDL-C levels in HNF1A-MODY patients.

| **Dependent Variable** | **Domain** | **Mean difference** | ***p*-value** | **95%Confidence Interval** | |
| --- | --- | --- | --- | --- | --- |
|  |  |  |  | **Lower** | **Upper** |
| LDL-C (mmol/L) | dimerization domain | 0.285 | 0.465 | -0.489 | 1.059 |
|  | DNA-binding domain | -0.554 | 0.015* | -0.999 | -0.110 |
|  | transactivation domain | 0.026 | 0.898 | -0.374 | 0.426 |

Abbreviations: *: *p* < 0.05, after adjusting for age of onset, gender, history of smoking and drinking, body mass index, glycated hemoglobin, triglyceride, and lipid-lowering therapy.

Analysis of covariance of TC levels in HNF1A-MODY patients.

| **Dependent Variable** | **Domain** | **Mean difference** | ***p*-value** | **95%Confidence Interval** | |
| --- | --- | --- | --- | --- | --- |
|  |  |  |  | **Lower** | **Upper** |
| TC (mmol/L) | dimerization domain | 0.515 | 0.216 | -0.307 | 1.337 |
|  | DNA-binding domain | -0.209 | 0.362 | -0.664 | 0.245 |
|  | transactivation domain | 0.169 | 0.439 | -0.263 | 0.601 |

Abbreviations: *: *p* < 0.05, after adjusting for age of onset, gender, body mass index, history of smoking and drinking, glycated hemoglobin, triglyceride, and lipid-lowering therapy.

**Supplementary Table 4** Sensitivity analysis of ANCOVA in HNF1A-MODY patients.

| **Dependent Variable** | **Domain** | **Mean difference** | ***p*-value** | **95%Confidence Interval** | |
| --- | --- | --- | --- | --- | --- |
|  |  |  |  | **Lower** | **Upper** |
| FCP (ng/ml) | dimerization domain | -0.797 | 0.048* | -1.586 | -0.008 |
|  | DNA-binding domain | -0.411 | 0.025* | -0.770 | -0.053 |
|  | transactivation domain | -0.256 | 0.182 | -0.635 | 0.123 |

Abbreviations: *: *p* < 0.05, after excluding clinically atypical cases (early or sustained insulin dependence), and adjusting for age of onset, gender, family history, duration of diabetes, body mass index, glycated hemoglobin, TyG index, and prior antidiabetic therapies.

| **Dependent Variable** | **Domain** | **Mean difference** | ***p*-value** | **95%Confidence Interval** | |
| --- | --- | --- | --- | --- | --- |
|  |  |  |  | **Lower** | **Upper** |
| FCP (ng/ml) | dimerization domain | -0.755 | 0.001* | -1.201 | -0.309 |
|  | DNA-binding domain | -0.333 | 0.038* | -0.648 | -0.018 |
|  | transactivation domain | -0.068 | 0.674 | -0.390 | 0.253 |

Abbreviations: *: *p* < 0.05, after adjusting for age of onset, family history, duration of diabetes, body mass index, glycated hemoglobin, TyG index, and antidiabetic therapies, with gender excluded from the model.

| **Dependent Variable** | **Domain** | **Mean difference** | ***p*-value** | **95%Confidence Interval** | |
| --- | --- | --- | --- | --- | --- |
|  |  |  |  | **Lower** | **Upper** |
| FCP (ng/ml) | dimerization domain | -0.771 | <0.001* | -1.205 | -0.337 |
|  | DNA-binding domain | -0.336 | 0.032* | -0.643 | -0.030 |
|  | transactivation domain | -0.080 | 0.600 | -0.382 | 0.222 |

Abbreviations: *: *p* < 0.05, after adjusting for gender, duration of diabetes, body mass index, glycated hemoglobin, TyG index, and antidiabetic therapies, with age of onset and family history excluded from the model.

| **Dependent Variable** | **Domain** | **Mean difference** | ***p*-value** | **95%Confidence Interval** | |
| --- | --- | --- | --- | --- | --- |
|  |  |  |  | **Lower** | **Upper** |
| FCP (ng/ml) | dimerization domain | -0.714 | 0.002* | -1.160 | -0.267 |
|  | DNA-binding domain | -0.283 | 0.075 | -0.595 | 0.029 |
|  | transactivation domain | -0.020 | 0.904 | -0.342 | 0.303 |

Abbreviations: *: *p* < 0.05, after adjusting for age of onset, gender, family history, duration of diabetes, body mass index, and antidiabetic therapies, with glycated hemoglobin and TyG index excluded from the model.

| **Dependent Variable** | **Domain** | **Mean difference** | ***p*-value** | **95%Confidence Interval** | |
| --- | --- | --- | --- | --- | --- |
|  |  |  |  | **Lower** | **Upper** |
| FCP (ng/ml) | dimerization domain | -0.737 | 0.002* | -1.191 | -0.283 |
|  | DNA-binding domain | -0.280 | 0.083 | -0.597 | 0.037 |
|  | transactivation domain | 0.001 | 0.996 | -0.321 | 0.322 |

Abbreviations: *: *p* < 0.05, after adjusting for age of onset, gender, family history, duration of diabetes, body mass index, glycated hemoglobin, and TyG index, with antidiabetic therapies excluded from the model.

| **Dependent Variable** | **Domain** | **Mean difference** | ***p*-value** | **95%Confidence Interval** | |
| --- | --- | --- | --- | --- | --- |
|  |  |  |  | **Lower** | **Upper** |
| LDL-C (mmol/L) | dimerization domain | -0.046 | 0.948 | -1.435 | 1.344 |
|  | DNA-binding domain | -0.399 | 0.120 | -0.906 | 0.107 |
|  | transactivation domain | 0.055 | 0.818 | -0.422 | 0.532 |

Abbreviations: *: *p* < 0.05, after excluding clinically atypical cases (early or sustained insulin dependence), and adjusting for age of onset, gender, history of smoking and drinking, body mass index, glycated hemoglobin, triglyceride, and lipid-lowering therapy.

| **Dependent Variable** | **Domain** | **Mean difference** | ***p*-value** | **95%Confidence Interval** | |
| --- | --- | --- | --- | --- | --- |
|  |  |  |  | **Lower** | **Upper** |
| LDL-C (mmol/L) | dimerization domain | 0.283 | 0.406 | -0.392 | 0.959 |
|  | DNA-binding domain | -0.451 | 0.033* | -0.865 | -0.037 |
|  | transactivation domain | 0.001 | 0.998 | -0.375 | 0.376 |

Abbreviations: *: *p* < 0.05, after adjusting for age of onset, gender, history of smoking and drinking, body mass index, glycated hemoglobin, and triglyceride, with lipid-lowering therapy excluded from the model.

| **Dependent Variable** | **Domain** | **Mean difference** | ***p*-value** | **95%Confidence Interval** | |
| --- | --- | --- | --- | --- | --- |
|  |  |  |  | **Lower** | **Upper** |
| LDL-C (mmol/L) | dimerization domain | 0.738 | 0.069 | -0.059 | 1.535 |
|  | DNA-binding domain | -0.687 | 0.004* | -1.151 | -0.224 |
|  | transactivation domain | 0.057 | 0.783 | -0.354 | 0.468 |

Abbreviations: *: *p* < 0.05, after adjusting for age of onset, gender, history of smoking and drinking, body mass index, and lipid-lowering therapy, with glycated hemoglobin and triglyceride excluded from the model.

| **Dependent Variable** | **Domain** | **Mean difference** | ***p*-value** | **95%Confidence Interval** | |
| --- | --- | --- | --- | --- | --- |
|  |  |  |  | **Lower** | **Upper** |
| LDL-C (mmol/L) | dimerization domain | 0.127 | 0.723 | -0.583 | 0.837 |
|  | DNA-binding domain | -0.585 | 0.010* | -1.026 | -0.144 |
|  | transactivation domain | 0.030 | 0.882 | -0.369 | 0.429 |

Abbreviations: *: *p* < 0.05, after adjusting for gender, history of smoking, body mass index, glycated hemoglobin, triglyceride, and lipid-lowering therapy, with age of onset and history of drinking excluded from the model.

| **Dependent Variable** | **Domain** | **Mean difference** | ***p*-value** | **95%Confidence Interval** | |
| --- | --- | --- | --- | --- | --- |
|  |  |  |  | **Lower** | **Upper** |
| LDL-C (mmol/L) | dimerization domain | 0.063 | 0.870 | -0.702 | 0.828 |
|  | DNA-binding domain | -0.651 | 0.005* | -1.098 | -0.203 |
|  | transactivation domain | -0.020 | 0.919 | -0.415 | 0.375 |

Abbreviations: *: *p* < 0.05, after adjusting for age of onset, gender, history of smoking and drinking, glycated hemoglobin, triglyceride, and lipid-lowering therapy, with body mass index excluded from the model.

| **Dependent Variable** | **Domain** | **Mean difference** | ***p*-value** | **95%Confidence Interval** | |
| --- | --- | --- | --- | --- | --- |
|  |  |  |  | **Lower** | **Upper** |
| HDL-C (mmol/L) | dimerization domain | 0.139 | 0.490 | -0.262 | 0.541 |
|  | DNA-binding domain | 0.153 | 0.046* | 0.003 | 0.303 |
|  | transactivation domain | 0.089 | 0.209 | -0.051 | 0.230 |

Abbreviations: *: *p* < 0.05, after excluding clinically atypical cases (early or sustained insulin dependence), and adjusting for age of onset, gender, history of smoking and drinking, glycated hemoglobin, triglyceride, total cholesterol, and lipid-lowering therapy.

| **Dependent Variable** | **Domain** | **Mean difference** | ***p*-value** | **95%Confidence Interval** | |
| --- | --- | --- | --- | --- | --- |
|  |  |  |  | **Lower** | **Upper** |
| HDL-C (mmol/L) | dimerization domain | 0.126 | 0.415 | -0.180 | 0.431 |
|  | DNA-binding domain | 0.232 | 0.012* | 0.052 | 0.411 |
|  | transactivation domain | 0.079 | 0.338 | -0.085 | 0.244 |

Abbreviations: *: *p* < 0.05, after adjusting for age of onset, gender, history of smoking and drinking, glycated hemoglobin, total cholesterol, and lipid-lowering therapy, with triglyceride excluded from the model.

| **Dependent Variable** | **Domain** | **Mean difference** | ***p*-value** | **95%Confidence Interval** | |
| --- | --- | --- | --- | --- | --- |
|  |  |  |  | **Lower** | **Upper** |
| HDL-C (mmol/L) | dimerization domain | 0.031 | 0.839 | -0.270 | 0.332 |
|  | DNA-binding domain | 0.313 | <0.001* | 0.150 | 0.476 |
|  | transactivation domain | 0.086 | 0.324 | -0.086 | 0.257 |

Abbreviations: *: *p* < 0.05, after adjusting for age of onset, gender, glycated hemoglobin, triglyceride, total cholesterol, and lipid-lowering therapy, with history of smoking and drinking excluded from the model.

| **Dependent Variable** | **Domain** | **Mean difference** | ***p*-value** | **95%Confidence Interval** | |
| --- | --- | --- | --- | --- | --- |
|  |  |  |  | **Lower** | **Upper** |
| HDL-C (mmol/L) | dimerization domain | 0.192 | 0.156 | -0.075 | 0.458 |
|  | DNA-binding domain | 0.282 | 0.001* | 0.115 | 0.449 |
|  | transactivation domain | 0.025 | 0.742 | -0.126 | 0.177 |

Abbreviations: *: *p* < 0.05, after adjusting for age of onset, gender, history of smoking and drinking, glycated hemoglobin, triglyceride, and total cholesterol, with lipid-lowering therapy excluded from the model.

| **Dependent Variable** | **Domain** | **Mean difference** | ***p*-value** | **95%Confidence Interval** | |
| --- | --- | --- | --- | --- | --- |
|  |  |  |  | **Lower** | **Upper** |
| HDL-C (mmol/L) | dimerization domain | 0.126 | 0.378 | -0.158 | 0.410 |
|  | DNA-binding domain | 0.230 | 0.011 | 0.054 | 0.406 |
|  | transactivation domain | 0.079 | 0.329 | -0.081 | 0.239 |

Abbreviations: *: *p* < 0.05, after adjusting for gender, history of smoking, glycated hemoglobin, triglyceride, total cholesterol, and lipid-lowering therapy, with age of onset and history of drinking excluded from the model.

**Supplementary Table 5** Linear mixed model

| **Dependent Variable** | **Domain** | **Mean difference** | ***p*-value** | **95%Confidence Interval** | |
| --- | --- | --- | --- | --- | --- |
|  |  |  |  | **Lower** | **Upper** |
| FCP (ng/ml) | dimerization domain | -0.776 | 0.003* | -1.272 | -0.279 |
|  | DNA-binding domain | -0.399 | 0.022* | -0.737 | -0.060 |
|  | transactivation domain | -0.064 | 0.717 | -0.416 | 0.287 |

Abbreviations: *: *p* < 0.05, a linear mixed model was fitted with the mutation domain as a fixed effect and data source as a random intercept. Covariates adjusted for included age of onset, gender, family history, duration of diabetes, glycated hemoglobin, TyG index, and prior antidiabetic therapies.

| **Dependent Variable** | **Domain** | **Mean difference** | ***p*-value** | **95%Confidence Interval** | |
| --- | --- | --- | --- | --- | --- |
|  |  |  |  | **Lower** | **Upper** |
| LDL-C (mmol/L) | dimerization domain | 0.285 | 0.465 | -0.489 | 1.059 |
|  | DNA-binding domain | -0.554 | 0.015* | -0.999 | -0.110 |
|  | transactivation domain | 0.026 | 0.898 | -0.374 | 0.426 |

Abbreviations: *: *p* < 0.05, a linear mixed model was fitted with the mutation domain as a fixed effect and data source as a random intercept. Covariates adjusted for included age of onset, gender, body mass index, history of smoking and drinking, glycated hemoglobin, triglyceride, and lipid-lowering therapy.

| **Dependent Variable** | **Domain** | **Mean difference** | ***p*-value** | **95%Confidence Interval** | |
| --- | --- | --- | --- | --- | --- |
|  |  |  |  | **Lower** | **Upper** |
| HDL-C (mmol/L) | dimerization domain | 0.111 | 0.468 | -0.193 | 0.416 |
|  | DNA-binding domain | 0.224 | 0.015* | 0.045 | 0.403 |
|  | transactivation domain | 0.075 | 0.366 | -0.089 | 0.238 |

Abbreviations: *: *p* < 0.05, a linear mixed model was fitted with the mutation domain as a fixed effect and data source as a random intercept. Covariates adjusted for included age of onset, gender, smoking and drinking history, glycated hemoglobin, triglyceride, total cholesterol, and lipid-lowering therapy.

# References

1. Ding Y, Zhang Q, Gao S, Li J, Chang G, Wang Y, et al. Focusing on Rare Variants Related to Maturity-Onset Diabetes of the Young in Children. *Pediatr Diabetes* (2025) 2025:8155443. doi:10.1155/pedi/8155443

2. Zhenjing W, Caihui Q, Mingzhong T, Xin L, Xin L, Chao X, et al. Clinical phenotype and genetic analysis of MODY3 caused by the HNF1A-c.47T>A variant. *Chin J Endocrinol Metab* (2025) 41(8):643-8. doi:10.3760/cma.j.cn311282-20250429-00225

3. Fangnan C, Qianyue Y, Yue J. One case report of MODY 3 by hepatocyte nuclear factor1α gene mutation. *Chin J School Doctor* (2023) 37(05):396-8. doi:10.20161/j.cnki.32-1199/r.2023.05.022

4. Ying S, Qian C, Gang Y, Xuefeng Y, Wentao H. A case of a monogenic diabetic MODY3 family. *J Clin Inter Med* (2022) 39(10):709-10. doi:10.3969/j.issn.1001-9057.2022.10.019

5. Zhang Y, Jiang Y, Li W, Li X. A case of adult-onset diabetes type 3 in an adolescent. *J Clin Inter Med* (2021) 38(10):701-3. doi:10.3969/j.issn.1001-9057.2021.10.017

6. Ren X, Liu M, Yan C, Zhang S. HNF-1α gene mutation in a family with MODY3 diabetes mellitus and literature review. *Chin J Diffic and Compl Cas* (2021) 20(08):838-40. doi:10.3969/j.issn.1671-6450.2021.08.018

7. Junying S, Xiaoming S. A Case of Maturity-Onset Diabetes of the Young with Hearing Impairment and Literature Review. *Chinese Journal of Rural Medicine and Pharmacy* (2020) 27(24):41. doi:10.19542/j.cnki.1006-5180.004681

8. Kang X, Ding W, Chen X, Yong Z, Ke Z, Zhibin W, et al. Clinical characteristics and HNF1ɑ gene analysis of a family with early-onset diabetes mellitus. *Jouranl of prectical diabetology* (2017) 13(03):36-9.

9. Zhang Y, Li L, Luo X. Gene mutation analysis of a case of type 3 "juvenile-onset adult-onset diabetes mellitus". *China Medical Engineering* (2015) 23(09):205-6.

10. Enjing N, Bingzi D, Fangchao L, Yingchao W, Yangang W, Zhengju F. GENE DETECTION OF MATURITY-ONSET DIABETES OF THE YOUNG WITH KETOSIS AS THE INITIAL SYMPTOM AND DISEASE ONSET IN ADOLESCENCE. *J Precis Med* (2019) 34(05):412-5+20. doi:10.13362/j.jpmed.201905009

11. Yanli W, Ruishuai Z. A case of adult-onset diabetes type 3 caused by HNF1A gene mutation in adolescent onset. *CHINESE MEDICAL CASE REPOSITORY* (2025) 07(01). doi:10.3760/cma.j.cmcr20250212-00488

12. Jing X, Yunchuan D, Shengliang W, Huijie Z, Hua S. Drug therapy in a family with maturity onset diabetes of young type 3: a case report. *Chin J Diabetes Mellitus* (2024) 16(05):556-60. doi:10.3760/cma.j.cn115791-20230909-00118

13. Yupeng L, Yonghua B, Ming L. Hepatic nuclear factor 1A-maturity-onset diabetes of the young patient with strong type 2 diabetes genetic background: a case report. *Chin J Diabetes Mellitus* (2024) 16(02):245-8. doi:10.3760/cma.j.cn115791-20230913-00122

14. Jie F, Pingping Z, Yueying F, Shuxia D, Lulu Y, Haibo L. Clinical characteristics and genetic analysis of children and adolescents with monogenic diabetes. *Chin J Med Genet* (2024) 41(7):783-9. doi:10.3760/cma.j.cn511374-20230515-00844

15. Jinxuan H, Yabin L, Yijie Q, Zhe L, Hongxia W, Xifeng Y, et al. Von Hippel-Lindau syndrome combined with hepatocyte nuclear factor 1 homeobox A mutation: a case report. *Chin J Diabetes Mellitus* (2024) 16(12):1418-22. doi:10.3760/cma.j.cn115791-20240312-00111

16. Ke H, Shousen S, Yanfang Z. Genetic screening of maturity onset diabetes of the young and related clinical features. *IMHGN* (2022) 28(21):3050-4. doi:10.3760/cma.j.issn.1007-1245.2022.21.018

17. Xiaoman Z, Wenqiong X. A case of MODY3 complicated with Klinefelter syndrome. *Int J Endocrinol Metab* (2023) 43(02):145-8. doi:10.3760/cma.j.cn121383-20211215-12037

18. Huifang P, Wenbo Z, Jing W, Yan L, Jie L, Liujun F, et al. Rapid progression of retinopathy in maturity onset diabetes of the young type 3: a case report. *Chin J Diabetes Mellitus* (2022) 14(11):1297-300. doi:10.3760/cma.j.cn115791-20220521-00230

19. Yan Z, Meijun L, Min L, Qiao Z, Lixin S. A case report of middle-aged diagnosed maturity onset diabetes of the young type 3 caused by mutation of hepatocyte nuclear factor 1 homeobox A. *Chin J Diabetes Mellitus* (2022) 14(12):1465-8. doi:10.3760/cma.j.cn115791-20220922-00486

20. Yanwen S, Tianwei G, Shanmei S, Yan B, Dalong Z. Three cases of maturity onset diabetes of the young and literature review. *Int J Endocrinol Metab* (2021) 41(03):237-40. doi:10.3760/cma.j.cn121383-20200729-07076

21. Xin P, Xiaoli W, Qiuyue W. Clinical characteristics of a family with maturity onset diabetes of the young type 3. *Chin J Diabetes Mellitus* (2021) 13(05):498-500. doi:10.3760/cma.j.cn115791-20201203-00702

22. Mingwei S, Yanling L, Peijie D, Fang W, Lin Z, Guijun Q. Maturity-onset diabetes of the young type 3 caused by genetic mutation of hepatocyte nuclear factor-1α: One family report. *Chin J Endocrinol Metab* (2020) 36(07):603-6. doi:10.3760/cma.j.cn311282-20200302-00118

23. Kuang N, Hou J, Luo J, Li L, Zhao X. A Case Report of a New Locus of Maturity-Onset Diabetes of the Young Type 3 (MODY3) *Advances in Clinical Medicine* (2022) 12(8):7423-8. doi:10.12677/acm.2022.1281072

24. Zhaomin L. *Gene study and clinical characteristics analysis of a MODY3 family caused by a new mutation ofHNF1A gene*, INNER MONGOLIA MEDICAL UNIVERSITY; 2023.

25. Jingjing G. *Functional analysis of a family with MODY3 caused by a mutation in the HNF1A gene*, SOOCHOW UNIVERSITY; 2022.

26. Youye H. *Family Collection and Pathogenic Gene Screening ofMaturity-onset diabetes of the young*, Anhui Medical University; 2019.

27. Lei W. *Study and analysis of pathogenicity of adolescent adult onset diabetes mellitus caused by HNF1A gene mutation*, INNER MONGOLIA MEDICAL UNIVERSITY; 2024.

28. Tiantian Q. *The clinical and genetic characterization of three MODY families*, Central South University; 2022.

29. Rong L. *Molecular Genetic Characteristics and Clinical Manifestations in Patients with MODY3*, Kunming Medical University; 2021.

30. Li L, Gong L, Zheng A, Yang Q, Pu D, Zhang Y. Maturity-onset diabetes of the young type 3 complicated with type 5: A case report and literature review. *Zhong Nan Da Xue Xue Bao Yi Xue Ban* (2024) 49(6):848-55. doi:10.11817/j.issn.1672-7347.2024.230594

31. An Y, Shao H, Chen W, Liang D. Dapagliflozin in maturity-onset diabetes of the young with HNF1A mutation and diabetic nephropathy. *Int Urol Nephrol* (2025) 57(5):1649-51. doi:10.1007/s11255-024-04317-w

32. To C, Liu L, Satoskar RS, Thuluvath PJ. Diabetic Hepatosclerosis in a Woman with Maturity-Onset Diabetes of the Young Type 3. *Dig Dis Sci* (2022) 67(6):2688-90. doi:10.1007/s10620-021-07005-2

33. Wang X, Cheng W, Wang Z, Liu C, Deng A, Li J. Chinese carrier of the HNF1A p.Gln444fs variant exhibits enhanced response to sulfonylureas. *Heliyon* (2024) 10(15):e35112. doi:10.1016/j.heliyon.2024.e35112

34. Wen Q, Li Y, Shao H, Ma J, Lin Y, Sun Y, et al. Two case reports of maturity-onset diabetes of the young type 3 caused by the hepatocyte nuclear factor 1α gene mutation. *Open Med (Wars)* (2023) 18(1):20230705. doi:10.1515/med-2023-0705

35. Peng H, Li J, Wang Z. De novo HNF1A mutation of young maturity-onset diabetes 3 of a young girl-Case report. *BMC Endocr Disord* (2023) 23(1):38. doi:10.1186/s12902-023-01293-7

36. Chen Y, Zhao J, Li X, Xie Z, Huang G, Yan X, et al. Prevalence of maturity-onset diabetes of the young in phenotypic type 2 diabetes in young adults: a nationwide, multi-center, cross-sectional survey in China. *Chin Med J (Engl)* (2023) 136(1):56-64. doi:10.1097/cm9.0000000000002321

37. Ren XY, Xue MR, Yan ZL, Zhang SJ, Liu M, Li AZ. Clinical Characteristics and Gene Mutations of Two Families with MODY 3 in Inner Mongolia. *Pharmgenomics Pers Med* (2022) 15:1019-27. doi:10.2147/pgpm.S371141

38. Li TL, Ding HX, Zhao XL, Luo J, Chen K, Tang Z, et al. A Case Report of MODY(3) Combined with Intestinal Neuroendocrine Tumor. *Chin Med Sci J* (2022) 37(2):167-70. doi:10.24920/003997

39. Wang M, Shu H, Xie J, Huang Y, Wang K, Feng R, et al. An intron mutation of HNF1A causes abnormal splicing and impairs its activity as a transcription factor. *Mol Cell Endocrinol* (2022) 545:111575. doi:10.1016/j.mce.2022.111575

40. Xu Q, Kan CX, Hou NN, Sun XD. Novel HNF1A gene mutation in maturity-onset diabetes of the young: A case report. *World J Clin Cases* (2022) 10(6):1909-13. doi:10.12998/wjcc.v10.i6.1909

41. Ma Y, Gong S, Wang X, Cai X, Xiao X, Gu W, et al. New clinical screening strategy to distinguish HNF1A variant-induced diabetes from young early-onset type 2 diabetes in a Chinese population. *BMJ Open Diabetes Res Care* (2020) 8(1). doi:10.1136/bmjdrc-2019-000745

42. Fu J, Wang T, Zhai X, Xiao X. Primary hepatocellular adenoma due to biallelic HNF1A mutations and its co-occurrence with MODY 3: case-report and review of the literature. *Endocrine* (2020) 67(3):544-51. doi:10.1007/s12020-019-02138-x

43. Tang J, Tang CY, Wang F, Guo Y, Tang HN, Zhou CL, et al. Genetic diagnosis and treatment of a Chinese ketosis-prone MODY 3 family with depression. *Diabetol Metab Syndr* (2017) 9:5. doi:10.1186/s13098-016-0198-5

44. Zhang M, Wang T, Shi L, Yang Y. Hepatocyte nuclear factor-α genetic mutation in a Chinese pedigree with maturity-onset diabetes of the young (MODY3). *Diabetes Metab Res Rev* (2015) 31(7):767-70. doi:10.1002/dmrr.2678

45. Fang C, Huang J, Huang Y, Chen L, Chen X, Hu J. A novel nonsense mutation of the HNF1α in maturity-onset diabetes of the young type 3 in Asian population. *Diabetes Res Clin Pract* (2015) 109(2):e5-7. doi:10.1016/j.diabres.2015.05.026

46. Ng MC, Li JK, So WY, Critchley JA, Cockram CS, Bell GI, et al. Nature or nurture: an insightful illustration from a Chinese family with hepatocyte nuclear factor-1 alpha diabetes (MODY3). *Diabetologia* (2000) 43(6):816-8. doi:10.1007/s001250051382

47. Cao B, Liu M, Zhang Y, Chen J, Li X, Su C, et al. An effective preselection criterion for MODY with an increasingly positive genetic testing rate by NGS: results from two cohorts of Chinese children. *Am J Physiol Endocrinol Metab* (2022) 323(6):E529-e34. doi:10.1152/ajpendo.00171.2022

48. Ng MC, Cockburn BN, Lindner TH, Yeung VT, Chow CC, So WY, et al. Molecular genetics of diabetes mellitus in Chinese subjects: identification of mutations in glucokinase and hepatocyte nuclear factor-1alpha genes in patients with early-onset type 2 diabetes mellitus/MODY. *Diabet Med* (1999) 16(11):956-63. doi:10.1046/j.1464-5491.1999.00188.x

49. Jap TS, Wu YC, Chiou JY, Kwok CF. A novel mutation in the hepatocyte nuclear factor-1alpha/MODY3 gene in Chinese subjects with early-onset Type 2 diabetes mellitus in Taiwan. *Diabet Med* (2000) 17(5):390-3. doi:10.1046/j.1464-5491.2000.00285.x
